# Association of the nutritional metabolic risk index with metabolic dysfunction-associated steatotic liver disease in a sub-plateau population

**DOI:** 10.3389/fendo.2026.1952423

**Published:** 2026-09-30

**Authors:** Huanqiong Fan, Minjie He, Guosong Jiang, Chaozhong Li, Songbin Li, Tingyuan Zhou

**Affiliations:** 1 Department of Pharmacy, Foshan University Affiliated Third Hospital, Foshan, Guangdong, China; 2 Department of Health Management Center, First Affiliated Hospital of Kunming Medical University, Kunming, Yunnan, China; 3 Department of Pulmonary and Critical Care Medicine, Northeast Yunnan Central Hospital; Northeast Yunnan Hospital of Kunming Medical University, Northeast Yunnan Clinical School of Kunming Medical University, Zhaotong, Yunnan, China; 4 Kunming Medical University, Kunming, Yunnan, China; 5 Department of Cardiology, First Affiliated Hospital of Kunming Medical University, Kunming, China; 6 Department of Gastroenterology, Foshan University Affiliated Third Hospital, Foshan, Guangdong, China

**Keywords:** geriatric nutritional risk index, metabolic dysfunction-associated steatotic liver disease, nutritional metabolic risk index, triglyceride-glucose index, waist-height ratio

## Abstract

**Background:**

Metabolic dysfunction-associated steatotic liver disease (MASLD) is closely associated with metabolic, anthropometric, and nutritional characteristics. The Nutritional Metabolic Risk Index (NMRI) integrates these dimensions, but its association with MASLD remains unclear. We evaluated the association of the NMRI with prevalent MASLD and its discriminative performance in a sub-plateau population.

**Methods:**

This cross-sectional study included 1,551 adults undergoing health examinations at approximately 1,900 m. K-means clustering was used to identify metabolic–nutritional profiles. Associations of NMRI with prevalent MASLD were assessed using multivariable logistic regression and restricted cubic splines. Discriminative and incremental performance were evaluated using receiver operating characteristic curves, net reclassification improvement (NRI), integrated discrimination improvement (IDI), and decision curve analysis, with bootstrap internal validation. Weighted quantile sum (WQS) regression assessed the relative statistical contributions of NMRI components.

**Results:**

Three metabolic–nutritional profiles were identified. Compared with the favorable metabolic profile, the adiposity-predominant and unfavorable metabolic–nutritional profiles had higher odds of MASLD (adjusted ORs, 6.62 and 7.85, respectively; both P<0.001). NMRI was inversely associated with prevalent MASLD (adjusted OR per unit increase, 0.71; 95% CI, 0.68–0.74), with an approximately linear association. NMRI showed higher discrimination than its individual components. Adding NMRI to the basic model improved discrimination (AUC, 0.822), reclassification (NRI, 0.759; IDI, 0.164), and net benefit across most examined threshold probabilities. Bootstrap validation yielded an optimism-corrected AUC of 0.815. WQS analysis assigned larger weights to TyG and WHtR than to GNRI. Associations remained consistent across subgroup and sensitivity analyses.

**Conclusion:**

Higher NMRI was consistently associated with lower odds of prevalent MASLD and showed favorable within-sample discrimination in this sub-plateau population. These findings support further evaluation of NMRI as a parsimonious composite indicator integrating nutritional, metabolic, and anthropometric characteristics associated with MASLD.

## Introduction

Metabolic dysfunction-associated steatotic liver disease (MASLD) constitutes a major global public health challenge, affecting approximately 30% of the global adult population (1, 2). It is projected to become a leading cause of cirrhosis and is also associated with hepatocellular carcinoma and increased cardiovascular mortality (3, 4). MASLD is a complex systemic disorder in which hepatic lipid accumulation is closely linked to multiorgan metabolic dysfunction (5, 6). Populations living in sub-plateau regions (elevations of 1,500–2,500 m), such as the Yunnan–Guizhou Plateau, experience environmental conditions that may influence metabolic homeostasis (7, 8). Previous studies suggest that hypoxic exposure may modulate hypoxia-inducible factor signaling, oxidative stress, insulin sensitivity, lipid metabolism, and adipose distribution (9–11). However, whether these altitude-related factors materially influence MASLD-associated metabolic characteristics remains uncertain. Accordingly, sub-plateau altitude provides a relevant contextual setting in which to characterize the metabolic and nutritional features associated with MASLD.

Current evidence indicates that MASLD involves multiple interconnected metabolic, nutritional, and anthropometric domains (12, 13). The triglyceride–glucose (TyG) index is a validated surrogate marker of insulin resistance (14, 15). The Geriatric Nutritional Risk Index (GNRI), which combines serum albumin with the ratio of current to ideal body weight, is an albumin- and body-weight-based index originally developed to assess nutrition-related risk (16). The waist-to-height ratio (WHtR) reflects central adiposity (17). Although the relevance of these measures to MASLD has been individually documented, their combined characteristics have been less extensively evaluated. This question may be of particular interest in sub-plateau populations, where environmental conditions may influence metabolic homeostasis, although the extent to which altitude contributes to these metabolic characteristics remains uncertain.

The Nutritional Metabolic Risk Index (NMRI), a previously proposed composite index integrating GNRI, TyG, and WHtR, was initially evaluated in relation to all-cause and cardiovascular mortality (18). By combining nutritional, metabolic, and anthropometric information within a single measure, NMRI provides a parsimonious representation of these interrelated characteristics. However, its association with prevalent MASLD and its discriminative performance in sub-plateau populations remain insufficiently characterized.

Accordingly, in adults undergoing health examinations in Kunming at approximately 1,900 m, we evaluated the association of the previously proposed NMRI with prevalent MASLD and its within-sample discriminative performance. K-means clustering was used to explore statistically derived metabolic–nutritional profiles, WQS regression to characterize the relative statistical contributions of NMRI components, and sensitivity analyses to assess the consistency of the findings.

## Methods

### Study design and population

This retrospective cross-sectional study included adults who underwent routine health examinations at the First Affiliated Hospital of Kunming Medical University (Kunming, Yunnan Province, Southwest China) between November 2024 and March 2026. Kunming is located in a sub-plateau setting at an elevation of approximately 1,900 m. Altitude was considered a contextual characteristic of the study setting rather than an individually measured exposure. Inclusion criteria comprised: (1) age ≥ 18 years; and (2) availability of complete data for liver ultrasonography, anthropometric measurements, and critical laboratory parameters.

To minimize the potential contribution of alcohol-related hepatic steatosis and reduce alcohol-related confounding, participants with alcohol consumption >140 g/week for men or >70 g/week for women were excluded. These thresholds were based on the 2017 Asia–Pacific Working Party on Non-alcoholic Fatty Liver Disease guidelines (19) and were retained as a conservative study-specific criterion to focus the analysis on metabolic–nutritional characteristics associated with hepatic steatosis. Participants with other chronic liver diseases, including viral hepatitis, drug-induced liver injury, or autoimmune hepatitis, were also excluded.

After applying these strict criteria and excluding individuals with missing core analytical data (such as incomplete data necessary for calculation of NMRI), a final cohort of 1551 participants was enrolled for the present analysis. The detailed participant screening and selection process is illustrated in **Figure 1**.

**Figure 1:**
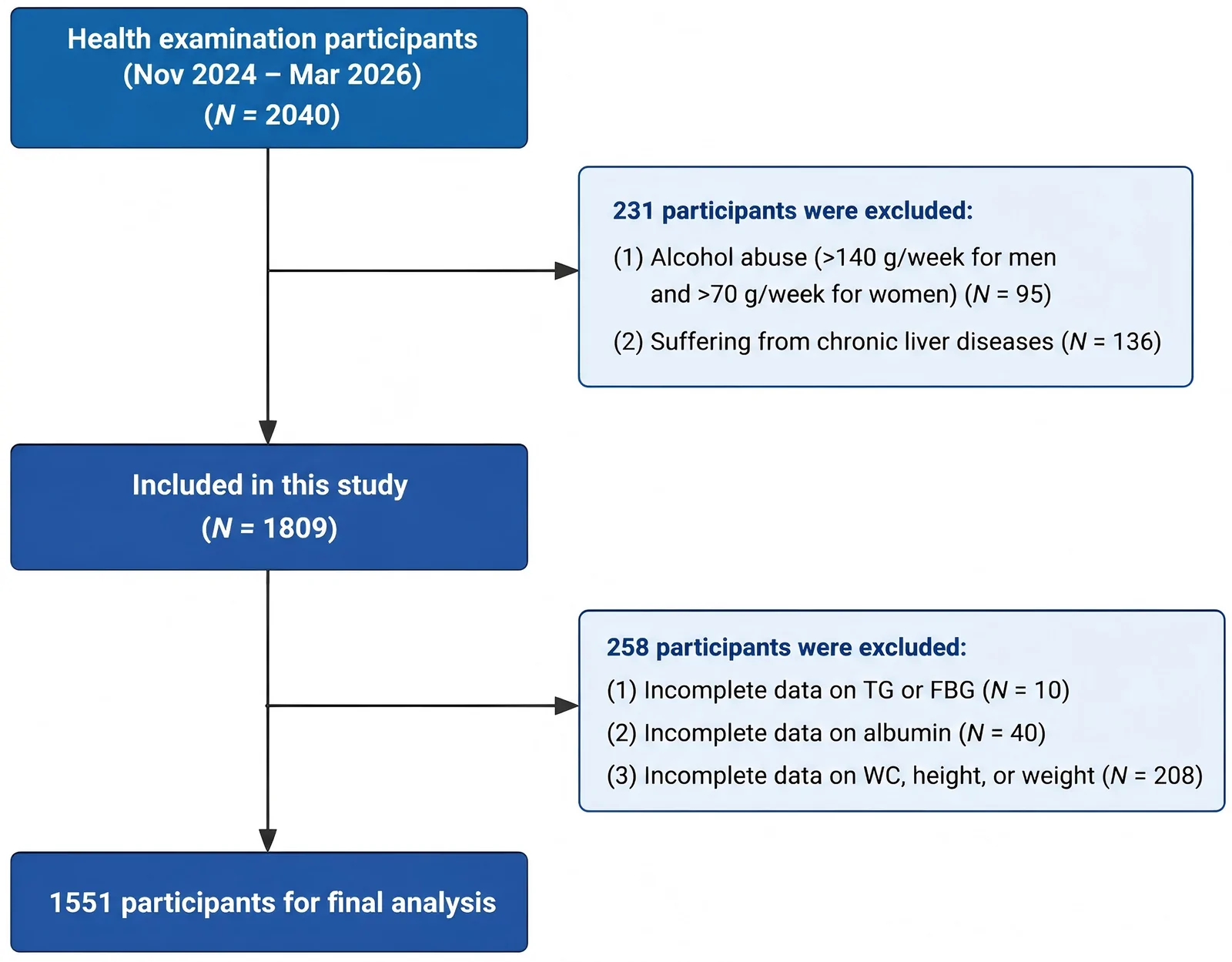
Flow diagram illustrating the participant selection process. TG, triglycerides; FBG, fasting blood glucose; WC, waist circumference.

The study protocol was approved by the Ethics Committee of the First Affiliated Hospital of Kunming Medical University (Approval No. 2026L215). Given the retrospective design of the study and the use of de-identified clinical data, the requirement for written informed consent was waived by the institutional review board.

### Demographic and anthropometric assessment

Physical examinations and face-to-face interviews were conducted by medically trained personnel using standardized questionnaires to collect demographic information (including geographic distribution), lifestyle habits, and medical history. Participants were measured for height, weight, and waist circumference barefoot and in light clothing, with the measurements recorded. Body mass index (BMI) was calculated as weight in kilograms divided by height in meters squared (kg/m^2^). Systolic blood pressure (SBP) and diastolic blood pressure (DBP) were measured using an automatic electronic sphygmomanometer (OMRON HBP-9031C). Prior to blood pressure assessment, participants were required to rest in a supine position for 5 to 10 minutes. Three consecutive measurements were obtained from the right brachial artery, and the mean value (in mmHg) was recorded for final analysis.

### Laboratory examination

Fasting venous blood samples (3 mL) were collected and processed within two hours using the Sysmex XN-9000 hematology analyzer (Sysmex, Japan) for complete blood count analysis. The institutional central laboratory conducted liver function and metabolic panel assessments, which included alanine aminotransferase (ALT), aspartate aminotransferase (AST), albumin, total cholesterol (TC), triglycerides (TG), high-density lipoprotein cholesterol (HDL-C), low-density lipoprotein cholesterol (LDL-C), creatinine, blood urea nitrogen (BUN), high-sensitivity C-reactive protein (hs-CRP), and fasting blood glucose (FBG), using the Roche cobas 8000 clinical chemistry analyzer (Roche, Switzerland).

### Exposure assessment

The formulas for the GNRI, TyG, WHtR, and NMRI were as follows: GNRI=1.489×albumin(g/L)+41.7×(actual weight/ideal weight), where ideal weight is

defined as height squared (m^2^) × 22; the ratio is set to 1 if actual weight exceeds ideal weight (18, 20–22).


TyG=ln[fasting triglycerides(mg/dL)×fasting glucose(mg/dL)/2]

WHtR=waist circumference(cm)/height(cm)

NMRI=GNRI/(TyG×WHtR)

### Definition of MASLD

Diagnosis of metabolic dysfunction-associated steatotic liver disease (MASLD) was based on the presence of hepatic steatosis on liver ultrasound together with at least one cardiometabolic risk factor, according to the international consensus definition of MASLD. Liver steatosis was identified by ultrasound findings (23). The primary required feature was diffusely increased near-field hepatic echogenicity exceeding that of the kidney and spleen, accompanied by progressive far-field acoustic attenuation—ultrasound signs that reflect increased hepatic fat content and its effect on sound propagation. At least one of the following additional ultrasound features was also required to support the diagnosis: (1) obscuration of intrahepatic vascular architecture; (2) mild to moderate hepatomegaly with rounded, blunted margins; (3) reduced Doppler flow signal intensity despite preservation of normal flow patterns; or (4) an indistinct right hepatic lobe contour. To enhance diagnostic reliability and reduce observer variability, diagnoses required agreement between two experienced sonographers; a third senior sonographer adjudicated any discrepant cases.

In addition to ultrasound-confirmed hepatic steatosis, participants were required to meet at least one of the following cardiometabolic criteria: (1) body mass index ≥23 kg/m^2^ for Asian individuals, or increased waist circumference, defined as >94 cm in men and >80 cm in women; (2) impaired glucose metabolism, defined as fasting serum glucose ≥5.6 mmol/L, 2-hour postprandial glucose ≥7.8 mmol/L, HbA1c ≥5.7%, a diagnosis of T2DM, or use of antidiabetic medications; (3) elevated blood pressure, defined as systolic/diastolic blood pressure ≥130/85 mmHg or use of antihypertensive medications; (4) elevated plasma triglycerides, defined as ≥1.70 mmol/L, or use of lipid-lowering treatment; or (5) low HDL-C, defined as ≤1.0 mmol/L in men and ≤1.3 mmol/L in women, or use of lipid-lowering treatment. Participants with ultrasound-confirmed hepatic steatosis who met at least one of these cardiometabolic criteria were classified as having MASLD (24).

### Covariate selection and definitions

The following covariates were collected for each enrolled participant:

(1) Demographic and lifestyle factors: age, sex, marital status (dichotomized as married versus other), smoking status, and drinking status (both coded as yes or no). (2) Anthropometric and clinical status: Current clinical status included physician-diagnosed hypertension and diabetes mellitus. Participants were classified as hypertensive if their measured systolic blood pressure (SBP) was ≥ 140 mmHg, diastolic blood pressure (DBP) was ≥ 90 mmHg, or if they reported a physician diagnosis. Diabetes was defined by any of the following criteria: fasting plasma glucose (FPG) ≥ 7.0 mmol/L (126 mg/dL), glycated hemoglobin (HbA1c) ≥ 6.5%, or a self-reported physician diagnosis of diabetes or hyperglycemia (25).(3) Biochemical parameters: ALT, AST, LDL-C, BUN, and hs-CRP.Variance inflation factors for the covariates are presented in **Supplementary Table S2**.

### Handling of missing data and data preprocessing

**Supplementary Table S1** summarizes the extent of missing data for each variable. The proportion of missing observations ranged from 0% to 13.86%. The missing-data mechanism was assessed using Little’s test for missing completely at random (MCAR). The test did not provide sufficient statistical evidence to reject the null hypothesis of MCAR (χ^2^ = 212.36, df=203, P = 0.312), indicating that the observed missingness pattern was compatible with, but did not establish, an MCAR mechanism. Multiple imputation was subsequently performed to reduce potential information loss associated with complete-case analysis, generating five completed datasets. The imputation model included key covariates and outcome variables, and estimates across the imputed datasets were combined using Rubin’s rules.

### Statistical analyses

K-means clustering based on Euclidean distance was used to identify metabolic–nutritional profiles. WHtR, TyG, and GNRI were standardized to z-scores before clustering (26). The optimal number of clusters was determined using the elbow method (**Supplementary Figure S1A**). As a sensitivity analysis, agglomerative hierarchical clustering was performed using the same variables, with the optimal cluster number selected by the elbow method (**Supplementary Figures S1B** and **S2**).

Participants were classified according to the presence or absence of MASLD. Continuous variables are presented as the mean ± standard deviation (SD) or median (interquartile range [IQR]), and categorical variables as frequencies (percentages). Appropriate parametric or nonparametric tests were applied for group comparisons.

Associations between metabolic–nutritional clusters, nutritional/metabolic indices, and MASLD were evaluated using multivariable logistic regression, with results expressed as odds ratios (ORs) and 95% confidence intervals (CIs). Covariates were selected *a priori* based on clinical knowledge and previously reported associations with MASLD and related metabolic characteristics. Three models were constructed using a sequential adjustment strategy: an unadjusted model; Model 1, adjusted for age, sex, marital status, smoking status, and drinking status; and Model 2, additionally adjusted for hypertension, diabetes, LDL-C, ALT, AST, BUN, and hs-CRP.

NMRI was analyzed both as a continuous variable and by tertiles, with trend tests performed by modeling tertiles as an ordinal variable. Restricted cubic spline analyses with four knots (5th, 35th, 65th, and 95th percentiles) were used to examine dose–response relationships and potential nonlinearity.

The discriminative performance of GNRI, TyG, WHtR, and NMRI for MASLD was assessed using receiver operating characteristic (ROC) curve analysis. Incremental discriminative value was evaluated using the net reclassification improvement (NRI), integrated discrimination improvement (IDI), and decision curve analysis (DCA), while differences in area under the curve (AUC) were compared using the DeLong test. To assess optimism in the discriminative performance of the multivariable model incorporating NMRI, internal validation was performed using 1,000 bootstrap resamples. In each resample, the model was refitted and its AUC was evaluated in both the bootstrap sample and the original dataset. Optimism was estimated as the mean difference between these AUCs, and the optimism-corrected AUC was calculated by subtracting the mean optimism from the apparent AUC.

Weighted quantile sum (WQS) regression with a binomial link was performed as an exploratory analysis to evaluate the joint association of TyG, WHtR, and GNRI with prevalent MASLD and their relative statistical contributions. All three components were entered in their original orientation without reverse coding and were transformed into quantile-based scores. A positive-direction WQS model was prespecified because the component-specific analyses indicated positive associations of TyG, WHtR, and GNRI with prevalent MASLD. Component weights were constrained to be non-negative and to sum to 1.

Prespecified subgroup analyses were conducted according to sex, age (<60 vs. ≥60 years), marital status, BMI (<24, 24–<28, and ≥28 kg/m^2^), hypertension, and diabetes. Associations within each subgroup were estimated using fully adjusted logistic regression models, and interactions were assessed using the Wald test.

Sensitivity analyses included complete-case analysis; recalculation of GNRI and NMRI using hemoglobin-corrected albumin to assess the potential influence of altitude-related hemoconcentration (27); exclusion of participants with extreme BMI (<18.5 or ≥35 kg/m^2^) or, separately, with hypertension or diabetes; and recalculation of GNRI and NMRI without truncating the actual weight/ideal weight ratio. To address potential structural overlap between NMRI components and the cardiometabolic criteria used in the primary MASLD definition, an alternative reduced-overlap outcome was defined as ultrasound-confirmed hepatic steatosis together with elevated blood pressure and/or low HDL-C, excluding adiposity-, glucose-, and triglyceride-based criteria. Associations with this alternative outcome and incremental discriminative performance were assessed using logistic regression and AUC, NRI, and IDI, respectively. Finally, hierarchical clustering was used as an alternative clustering approach to assess cross-method concordance with the K-means solution. Detailed results are provided in **Supplementary Tables S6**-**S13** and **Supplementary Figures S1**, **S2**.

All analyses were performed using R version 4.2.2 (R Foundation for Statistical Computing, Vienna, Austria) and Free Statistics version 2.1.1 (Beijing FreeClinical Medical Technology Co., Ltd., Beijing, China).

## Results

### Demographic and clinical characteristics

A total of 1,551 participants were included in the analysis, of whom 642 (41.39%) were diagnosed with MASLD, and 909 (58.61%) were without MASLD. The overall cohort comprised 42.94% females. Compared to the non-MASLD group, participants with MASLD were older and exhibited a higher proportion of males and were married. The MASLD group had a higher prevalence of smoking, alcohol consumption, hypertension, and diabetes (all P< 0.05). Metabolic and anthropometric differences included higher BMI (25.47 ± 2.85 vs 22.57 ± 2.56), fasting glucose (5.33 ± 1.40 vs 4.84 ± 1.15), triglycerides (median 1.98 vs 1.24 mmol/L), TyG index (9.10 ± 0.64 vs 8.49 ± 0.55), WHtR (0.52 ± 0.05 vs 0.47 ± 0.05), and GNRI (111.75 ± 3.68 vs 110.02 ± 4.12) in the MASLD group (all P< 0.001). HDL-C was lower, and liver enzymes (ALT, AST) were higher in MASLD (P< 0.001). LDL-C, BUN, and hs-CRP did not differ significantly between groups. A detailed summary of baseline characteristics for both groups was provided in **Table 1**.

**Table 1:** Baseline characteristics of individuals with and without MASLD.

Variables	Total (n = 1551)	Non-MASLD (n = 909)	MASLD (n = 642)	*P*-value
Age (years)	48.77 ± 12.19	47.77 ± 12.58	50.18 ± 11.48	< 0.001
Sex, n (%)				< 0.001
Female	666 (42.94)	479 (52.7)	187 (29.13)	
Male	885 (57.06)	430 (47.3)	455 (70.87)	
Marital status, n (%)				< 0.001
Other	145 (9.35)	106 (11.66)	39 (6.07)	
Married	1406 (90.65)	803 (88.34)	603 (93.93)	
Smoking status, n (%)				< 0.001
NO	1221 (78.72)	752 (82.73)	469 (73.05)	
Yes	330 (21.28)	157 (17.27)	173 (26.95)	
Drinking status, n (%)				< 0.001
NO	1056 (68.09)	672 (73.93)	384 (59.81)	
Yes	495 (31.91)	237 (26.07)	258 (40.19)	
Hypertension, n (%)				< 0.001
NO	1251 (80.66)	793 (87.24)	458 (71.34)	
Yes	300 (19.34)	116 (12.76)	184 (28.66)	
Diabetes, n (%)				< 0.001
NO	1451 (93.55)	870 (95.71)	581 (90.5)	
Yes	100 (6.45)	39 (4.29)	61 (9.5)	
BMI, (kg/m^2^)	23.77 ± 3.04	22.57 ± 2.56	25.47 ± 2.85	< 0.001
FBG (mmol/L)	5.04 ± 1.28	4.84 ± 1.15	5.33 ± 1.40	< 0.001
TC (mmol/L)	4.86 ± 0.95	4.80 ± 0.94	4.95 ± 0.96	0.003
TG (mmol/L)	1.51 (1.07, 2.20)	1.24 (0.93, 1.71)	1.98 (1.46, 2.95)	< 0.001
HDL-C (mmol/L)	1.29 ± 0.33	1.40 ± 0.33	1.13 ± 0.26	< 0.001
LDL-C (mmol/L)	2.92 ± 0.81	2.89 ± 0.80	2.95 ± 0.83	0.147
ALT (U/L)	20.10 (13.60, 30.67)	17.40 (12.00, 26.30)	25.15 (17.50, 36.00)	< 0.001
AST (U/L)	27.65 ± 9.78	26.71 ± 8.57	28.97 ± 11.15	< 0.001
Creatinine (μmol/L)	80.17 ± 16.64	79.19 ± 16.14	81.55 ± 17.24	0.006
BUN (mmol/L)	5.17 ± 1.27	5.14 ± 1.24	5.21 ± 1.30	0.325
hs-CRP (mg/L)	3.44 (0.92, 5.03)	3.49 (0.83, 5.01)	3.25 (1.01, 5.05)	0.415
GNRI, Mean ± SD	110.74 ± 4.03	110.02 ± 4.12	111.75 ± 3.68	< 0.001
TyG, Mean ± SD	8.75 ± 0.66	8.49 ± 0.55	9.10 ± 0.64	< 0.001
WHtR, Mean ± SD	0.49 ± 0.05	0.47 ± 0.05	0.52 ± 0.05	< 0.001

BMI, body mass index; FBG, fasting blood glucose; TC, total cholesterol; TG, triglycerides; HDL-C, high-density lipoprotein cholesterol; LDL-C, low-density lipoprotein cholesterol; ALT, alanine aminotransferase; AST, aspartate aminotransferase; BUN, blood urea nitrogen; hs-CRP, high-sensitivity C-reactive protein; GNRI, geriatric nutritional risk index; TyG, triglyceride-glucose index; WHtR, waist-height ratio; NMRI, nutritional metabolic risk index.

### Cluster analysis identified three metabolic–nutritional profiles

Cluster analysis identified three statistically derived metabolic–nutritional profiles (**Figure 2** and **Supplementary Table S3**). Cluster 1 was characterized by higher WHtR with intermediate TyG and lower GNRI levels and was termed the adiposity-predominant profile. Cluster 2 showed relatively higher TyG, WHtR, and GNRI levels and was termed the unfavorable metabolic–nutritional profile. Cluster 3 was characterized by lower WHtR and TyG levels, together with lower GNRI, and was termed the favorable metabolic profile. The three-cluster solution showed a clear clustering tendency (Hopkins statistic, 0.822) but modest separation (average silhouette coefficient, 0.240). K-means assignments showed substantial cross-method concordance with hierarchical clustering (overall agreement, 84.27%; Cohen’s κ=0.7624). These groups represent exploratory, statistically derived profiles illustrating heterogeneity in metabolic, anthropometric, and nutrition-related characteristics (**Supplementary Table S13**).

**Figure 2:**
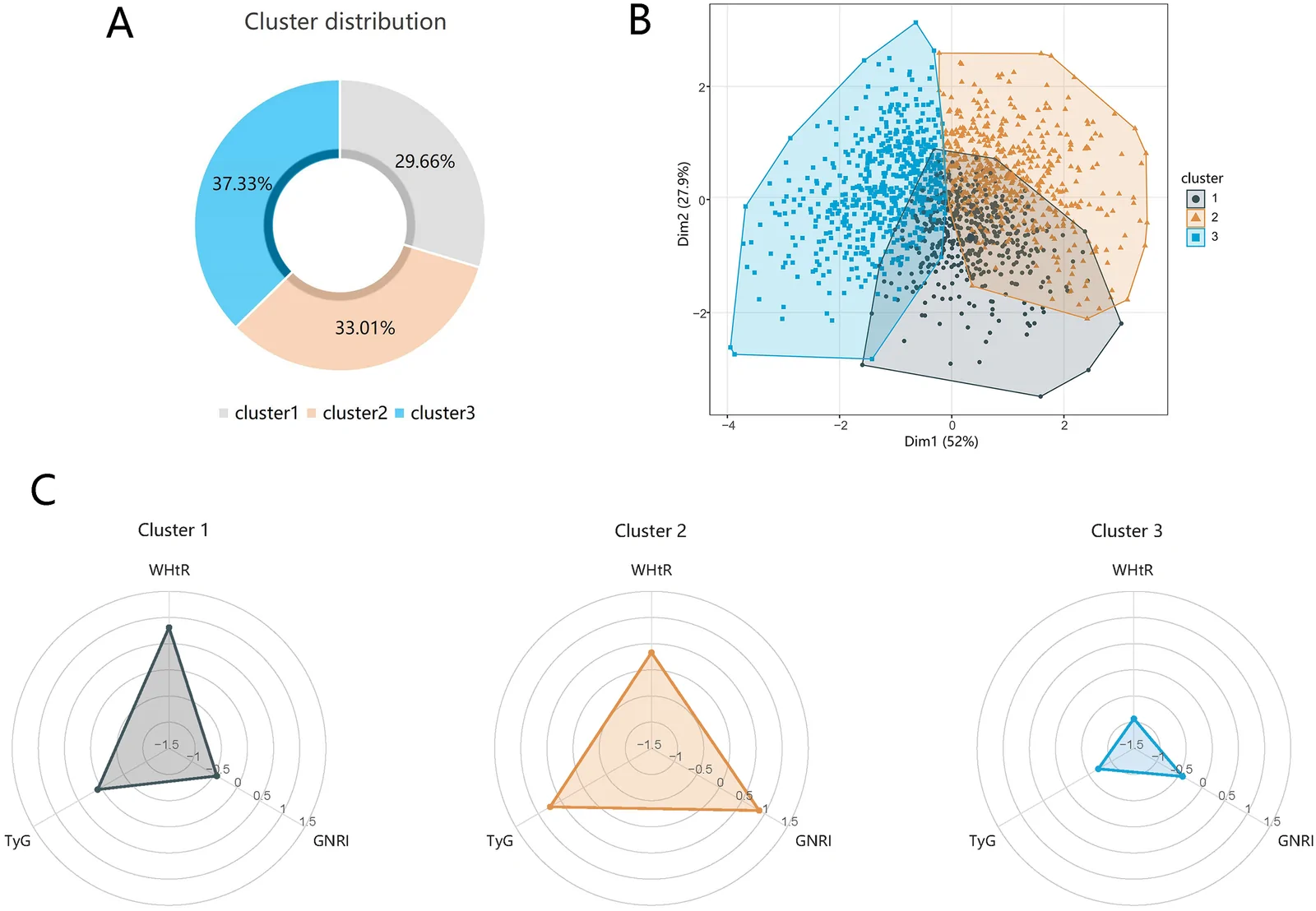
Visualization and clinical characteristics of the three clusters derived by k-means analysis. **(A)** Cluster distribution of participants. **(B)** Scatter plots. **(C)** Radar plots. Cluster 1 was characterized as an adiposity-predominant profile, Cluster 2 as an unfavorable metabolic–nutritional profile, and Cluster 3 as a favorable metabolic profile.

### Association of metabolic–nutritional clusters with MASLD

Cluster analysis identified three distinct metabolic–nutritional profiles associated with differential odds of MASLD (**Table 2**). Compared with Cluster 3, characterized by a favorable metabolic profile, Clusters 1 and 2 had markedly higher odds of MASLD. After full adjustment, the odds of MASLD remained significantly elevated in Cluster 1 (OR, 6.62; 95% CI, 4.77–9.18) and Cluster 2 (OR, 7.85; 95% CI, 5.67–10.87) (both P< 0.001). A significant trend in the odds of prevalent MASLD was observed across clusters (P for trend<0.001).

**Table 2:** Associations of the three metabolic-nutritional profiles with prevalent MASLD.

Exposure	Crude model		Model 1		Model 2	
	OR (95% CI)	*P-*value	OR (95% CI)	*P-*value	OR (95% CI)	*P-*value
Cluster 3	1(Ref)		1(Ref)		1(Ref)	
Cluster 1	8.46 (6.22~11.5)	<0.001	7.53 (5.47~10.37)	<0.001	6.62 (4.77~9.18)	<0.001
Cluster 2	11.64 (8.58~15.79)	<0.001	9.81 (7.18~13.41)	<0.001	7.85 (5.67~10.87)	<0.001
P for trend		<0.001		<0.001		<0.001

Data presented are ORs and 95% CIs. Model 1: adjusted for age, sex, marital status, smoking status, and drinking status. Model 2: adjusted for variables in Model 1 plus hypertension, diabetes, LDL-C, ALT, AST, BUN, and hs-CRP. MASLD, metabolic dysfunction-associated steatotic liver disease; LDL-C, low-density lipoprotein cholesterol; ALT, alanine aminotransferase; AST, aspartate aminotransferase; BUN, blood urea nitrogen; hs-CRP, high-sensitivity C-reactive protein. Cluster 1 was characterized as an adiposity-predominant profile, Cluster 2 as an unfavorable metabolic–nutritional profile, and Cluster 3 as a favorable metabolic profile.

### Association of the NMRI with MASLD

**Table 3** summarizes the associations between metabolic–nutritional indices and MASLD. In fully adjusted analyses (Model 2), higher GNRI, TyG, and WHtR levels were significantly associated with increased odds of MASLD (GNRI: OR, 1.09; 95% CI, 1.06–1.13; TyG: OR, 5.03; 95% CI, 3.96–6.40; WHtR per 0.1-unit increase: OR, 6.51; 95% CI, 4.91–8.63; all P< 0.001). In contrast, NMRI showed a strong inverse association with MASLD, with each unit increase in NMRI associated with lower odds of MASLD (OR, 0.71; 95% CI, 0.68–0.74; P< 0.001). When categorized into tertiles, participants in higher NMRI categories had progressively lower odds of MASLD compared with those in the lowest tertile (Q2: OR, 0.31; Q3: OR, 0.07; P for trend< 0.001). Restricted cubic spline analysis (**Figure 3**) demonstrated a linear inverse relationship between NMRI and odds of MASLD, with no significant evidence of non-linearity (P for non-linearity > 0.05).

**Table 3:** Associations of NMRI and its component indices with prevalent MASLD.

Exposure	Crude model		Model 1		Model 2	
	OR (95% CI)	*P-*value	OR (95% CI)	*P-*value	OR (95% CI)	*P-*value
GNRI	1.12 (1.09~1.15)	<0.001	1.12 (1.09~1.15)	<0.001	1.09 (1.06~1.13)	<0.001
TyG	6.03 (4.82~7.54)	<0.001	5.58 (4.43~7.04)	<0.001	5.03 (3.96~6.4)	<0.001
WHtR per 0.1 unit	8.25 (6.36~10.7)	<0.001	7.79 (5.92~10.26)	<0.001	6.51 (4.91~8.63)	<0.001
NMRI	0.7 (0.67–0.73)	<0.001	0.69 (0.66~0.72)	<0.001	0.71 (0.68~0.74)	<0.001
NMRI tertile						
T1	1(Ref)		1(Ref)		1(Ref)	
T2	0.3 (0.24~0.39)	<0.001	0.29 (0.22~0.38)	<0.001	0.31 (0.23~0.41)	<0.001
T3	0.06 (0.04~0.08)	<0.001	0.06 (0.04~0.08)	<0.001	0.07 (0.05~0.11)	<0.001
P for trend		<0.001		<0.001		<0.001

Data presented are ORs and 95% CIs. Model 1: adjusted for age, sex, marital status, smoking status, and drinking status. Model 2: adjusted for variables in Model 1 plus hypertension, diabetes, LDL-C, ALT, AST, BUN, and hs-CRP. MASLD, Metabolic dysfunction-associated steatotic liver disease; GNRI, geriatric nutritional risk index; TyG, Triglyceride-glucose index; WHtR, Waist-height ratio; NMRI, Nutritional Metabolic Risk Index; LDL-C, low-density lipoprotein cholesterol; ALT, alanine aminotransferase; AST, aspartate aminotransferase; BUN, blood urea nitrogen; hs-CRP, high-sensitivity C-reactive protein.

**Figure 3:**
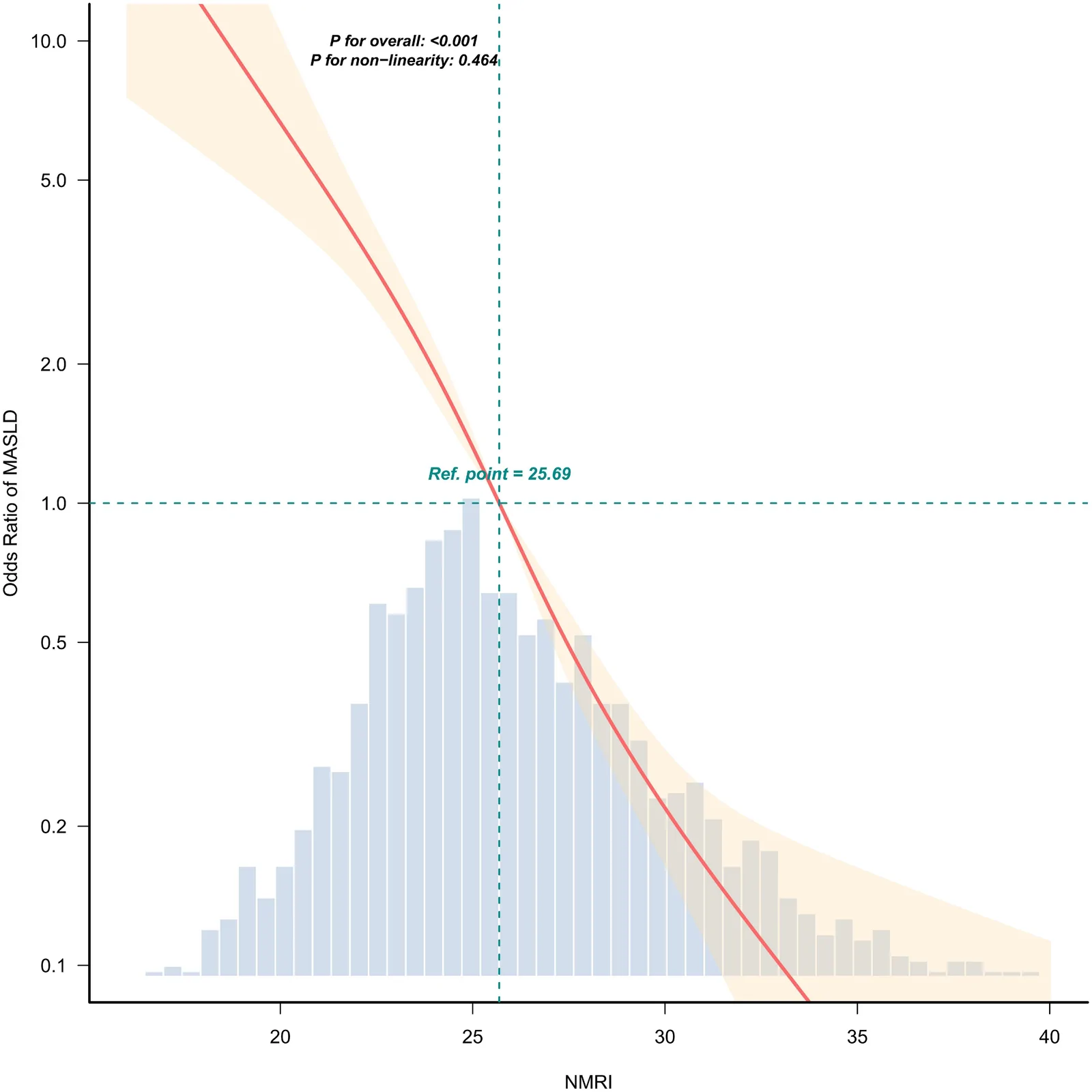
Dose-response associations between NMRI and MASLD based on RCS models. Models were adjusted for age, sex, marital status, smoking status, drinking status, hypertension, diabetes, LDL-C, ALT, AST, BUN, and hs-CRP. Reference point: Median. RCS: Restricted cubic spline. Reference: Ref. MASLD, Metabolic dysfunction-associated steatotic liver disease; NMRI, Nutritional metabolic risk index; LDL-C, low-density lipoprotein cholesterol; ALT, alanine aminotransferase; AST, aspartate aminotransferase; BUN, blood urea nitrogen; hs-CRP, high-sensitivity C-reactive protein.

### A comparative analysis of the discriminative values of the NMRI, GNRI, TyG, and WHtR indices for MASLD

ROC analysis showed that, among the individual indices, NMRI had the highest AUC for prevalent MASLD (0.800; 95% CI, 0.778–0.821), compared with TyG (0.774), WHtR (0.764), and GNRI (0.618) (**Figure 4**, **Supplementary Table S4**). When added to the basic model, NMRI improved the AUC to 0.822 (95% CI, 0.802–0.843), with corresponding improvements in NRI and IDI. However, simultaneous addition of GNRI, TyG, and WHtR yielded numerically higher discrimination and reclassification metrics than NMRI alone (AUC, 0.839 vs 0.822; NRI, 0.841 vs 0.759; IDI, 0.195 vs 0.164) (**Table 4**). These findings suggest that the potential value of NMRI lies in its parsimonious integration of metabolic, anthropometric, and nutritional-related information into a single composite measure rather than in superior discriminative performance over simultaneous modeling of its three components.

**Figure 4:**
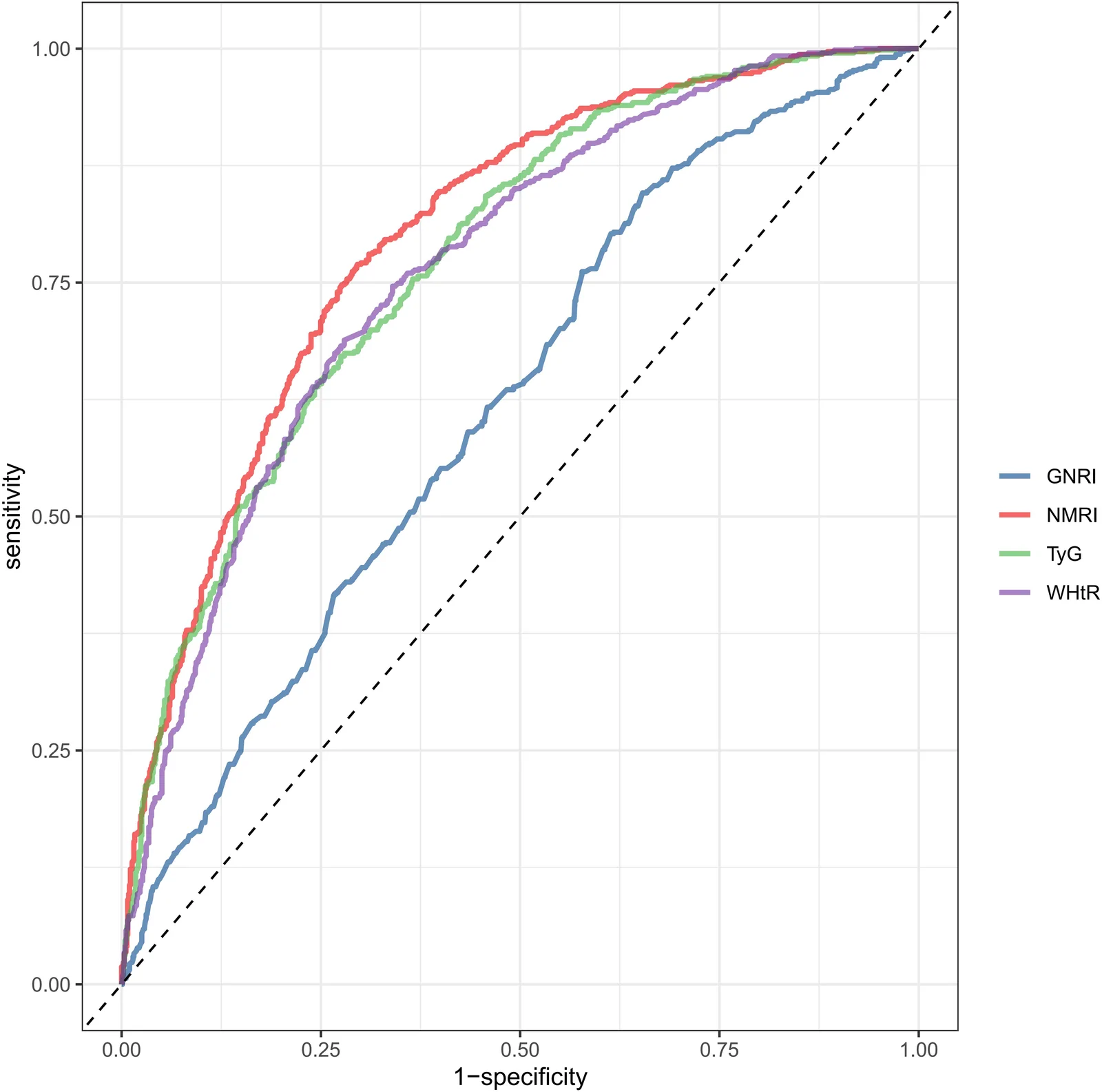
Receiver operating characteristic (ROC) curves of the NMRI, GNRI, TyG, and WHtR indices for MASLD. MASLD, metabolic dysfunction-associated steatotic liver disease; GNRI, geriatric nutritional risk index; TyG, triglyceride-glucose index; WHtR, waist-height ratio; NMRI, nutritional metabolic risk index.

**Table 4:** Incremental discriminative performance of NMRI and its component indices for prevalent MASLD.

Model	AUC (95% CI)	*P-*value	NRI (95% CI)	*P-*value	IDI (95% CI)	*P-*value
Basic model	0.720 (0.695, 0.745)		Ref		Ref	
+GNRI	0.732 (0.707, 0.757)	0.021	0.248(0.148,0.348)	<0.001	0.018(0.011,0.025)	<0.001
+TyG	0.805 (0.783, 0.826)	<0.001	0.667(0.572,0.763)	<0.001	0.129(0.112,0.147)	<0.001
+WHtR	0.797 (0.775, 0.819)	<0.001	0.638(0.542,0.734)	<0.001	0.116(0.099,0.132)	<0.001
+GNRI, TyG, WHtR	0.839 (0.819, 0.858)	<0.001	0.841(0.749,0.933)	<0.001	0.195(0.174,0.215)	<0.001
+NMRI	0.822 (0.802, 0.843)	<0.001	0.759(0.666,0.853)	<0.001	0.164(0.145,0.183)	<0.001

Basic models were adjusted for age, sex, marital status, smoking status, drinking status, hypertension, diabetes, LDL-C, ALT, AST, BUN, and hs-CRP. AUC, Area Under the Curve; NRI, Net reclassification improvement; IDI, Integrated discrimination improvement; GNRI, geriatric nutritional risk index; TyG, Triglyceride-glucose index; WHtR, Waist-height ratio; NMRI, Nutritional metabolic risk index.

Bootstrap internal validation with 1,000 resamples yielded an apparent AUC of 0.822 for the multivariable model incorporating NMRI. The mean bootstrap optimism was 0.007, resulting in an optimism-corrected AUC of 0.815. The modest difference between the apparent and optimism-corrected AUCs indicated limited optimism in the observed discriminative performance (**Supplementary Table S5**).

### Decision curve analysis of NMRI and its component indices for prevalent MASLD

Decision curve analysis showed that NMRI yielded a higher net benefit than the individual component indices across most of the examined threshold probabilities (**Figure 5**). TyG and WHtR also showed positive net benefits across a range of thresholds, whereas the net benefit of GNRI was comparatively limited. These findings characterize the decision-analytic performance of NMRI within the present study population and require confirmation in independent populations.

**Figure 5:**
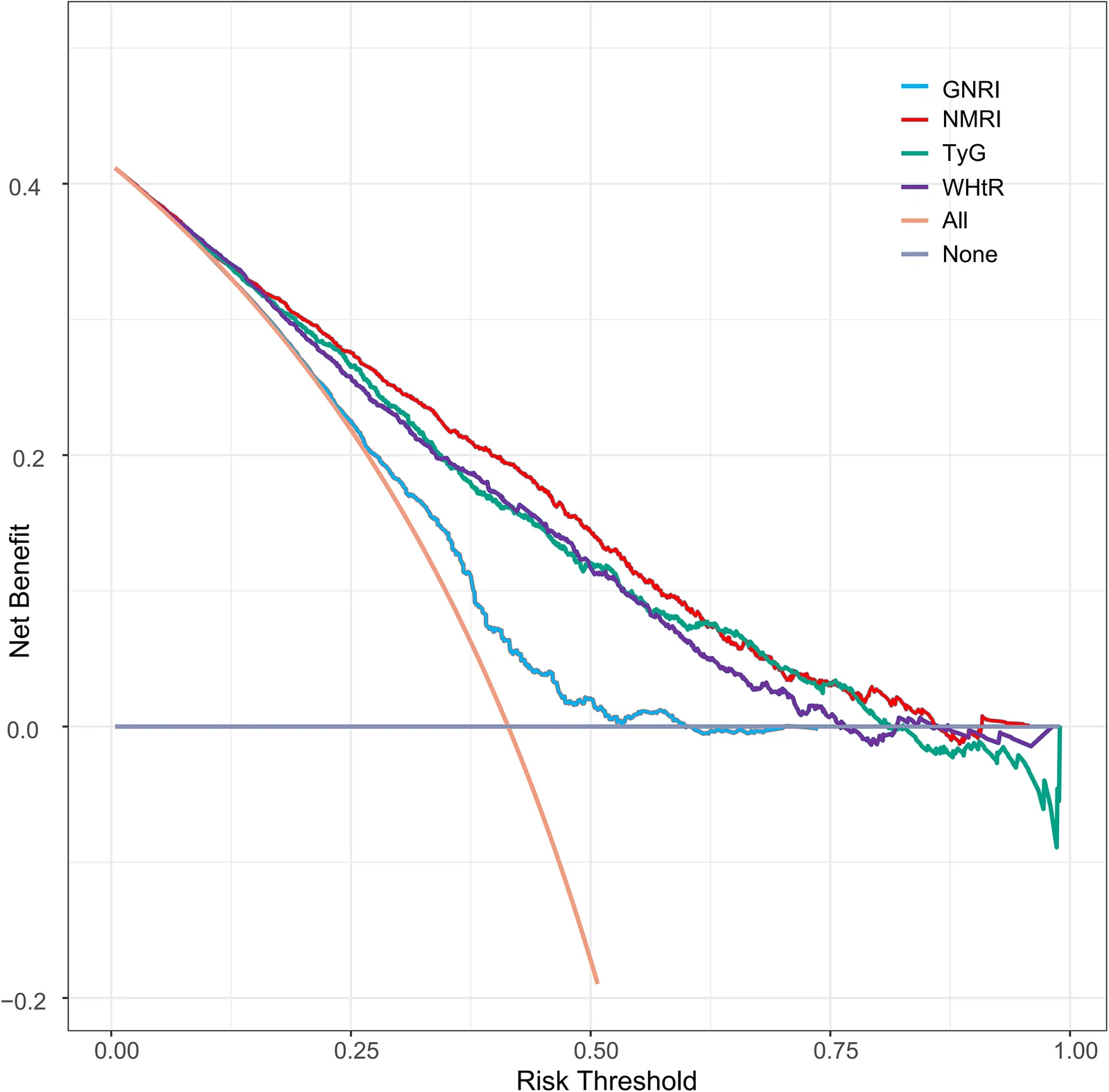
Decision curve analysis of NMRI, GNRI, TyG, and WHtR for prevalent metabolic dysfunction-associated steatotic liver disease (MASLD). NMRI showed a higher net benefit than the individual component indices across most of the examined threshold probabilities. MASLD, metabolic dysfunction-associated steatotic liver disease; NMRI, nutritional metabolic risk index; GNRI, geriatric nutritional risk index; TyG, triglyceride-glucose index; WHtR, waist-to-height ratio.

### WQS analyses

WQS regression assigned the largest weights to TyG (0.41) and WHtR (0.40), whereas GNRI received a smaller weight (0.19)(**Figure 6**). These weights represent the relative statistical contributions of the three components within the present dataset and should not be interpreted as direct measures of biological importance. Given the partial overlap of TyG- and WHtR-related variables with the primary MASLD definition, their relatively large weights may also partly reflect this structural overlap.

**Figure 6:**
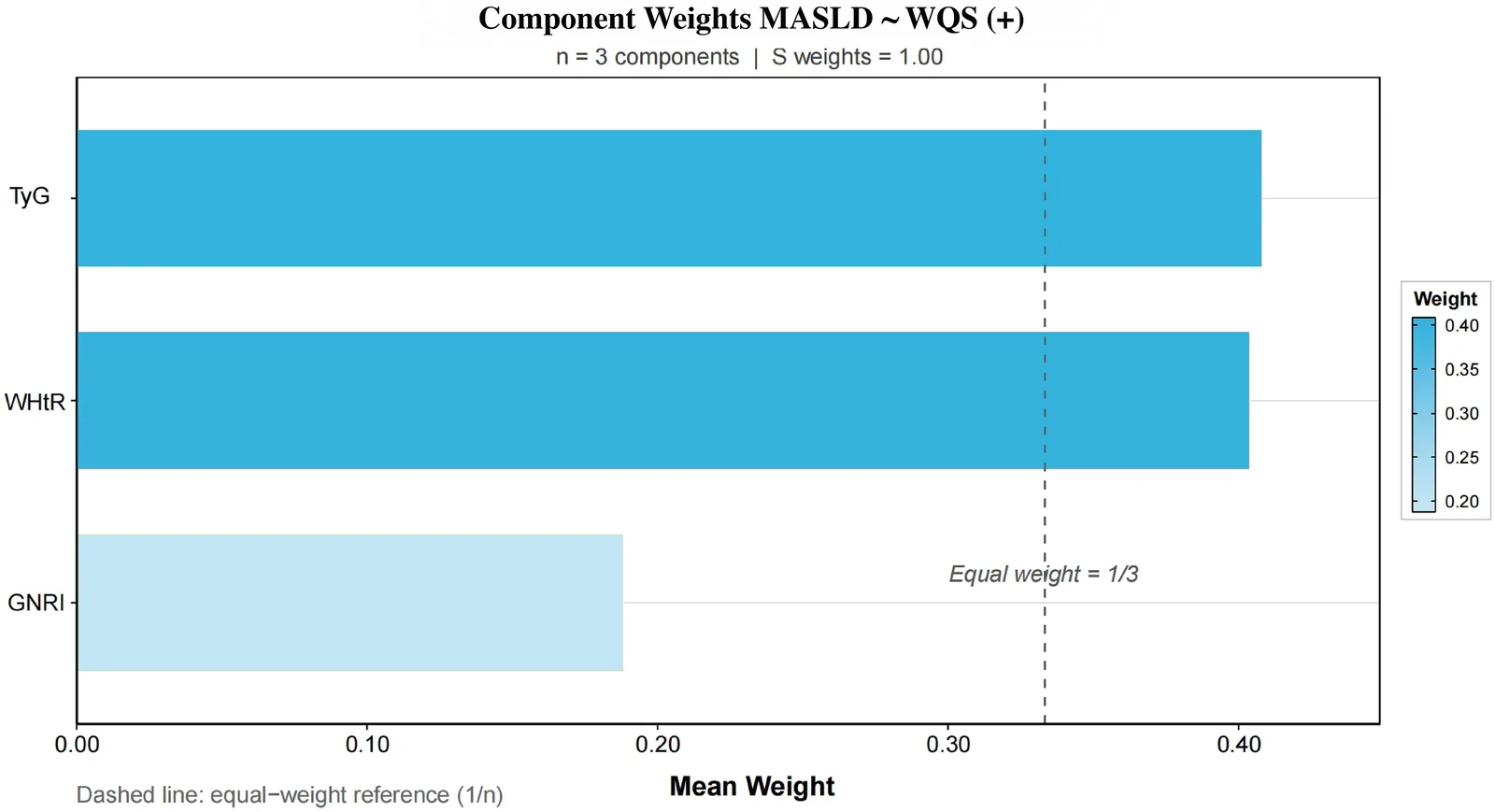
Relative weights of TyG, WHtR, and GNRI contributing to MASLD via WQS regression. WQS, weighted quantile sum; GNRI, geriatric nutritional risk index; TyG, triglyceride-glucose index; WHtR, waist-height ratio.

### Stratified associations of NMRI with MASLD

In the full sample, the adjusted model showed a statistically significant inverse association between the NMRI and MASLD (adjusted OR 0.71, 95% CI 0.68–0.74). Subgroup analyses demonstrated a consistent inverse association across age, marital status, BMI, hypertension, and diabetes strata. The association was nominally stronger in females (OR 0.67, 95% CI 0.62–0.73) than in males (OR 0.73, 95% CI 0.69–0.77), with evidence of heterogeneity by sex (*P* for interaction = 0.006). No other subgroup tested showed statistically significant interaction (all *P* interaction >0.05) (**Figure 7**).

**Figure 7:**
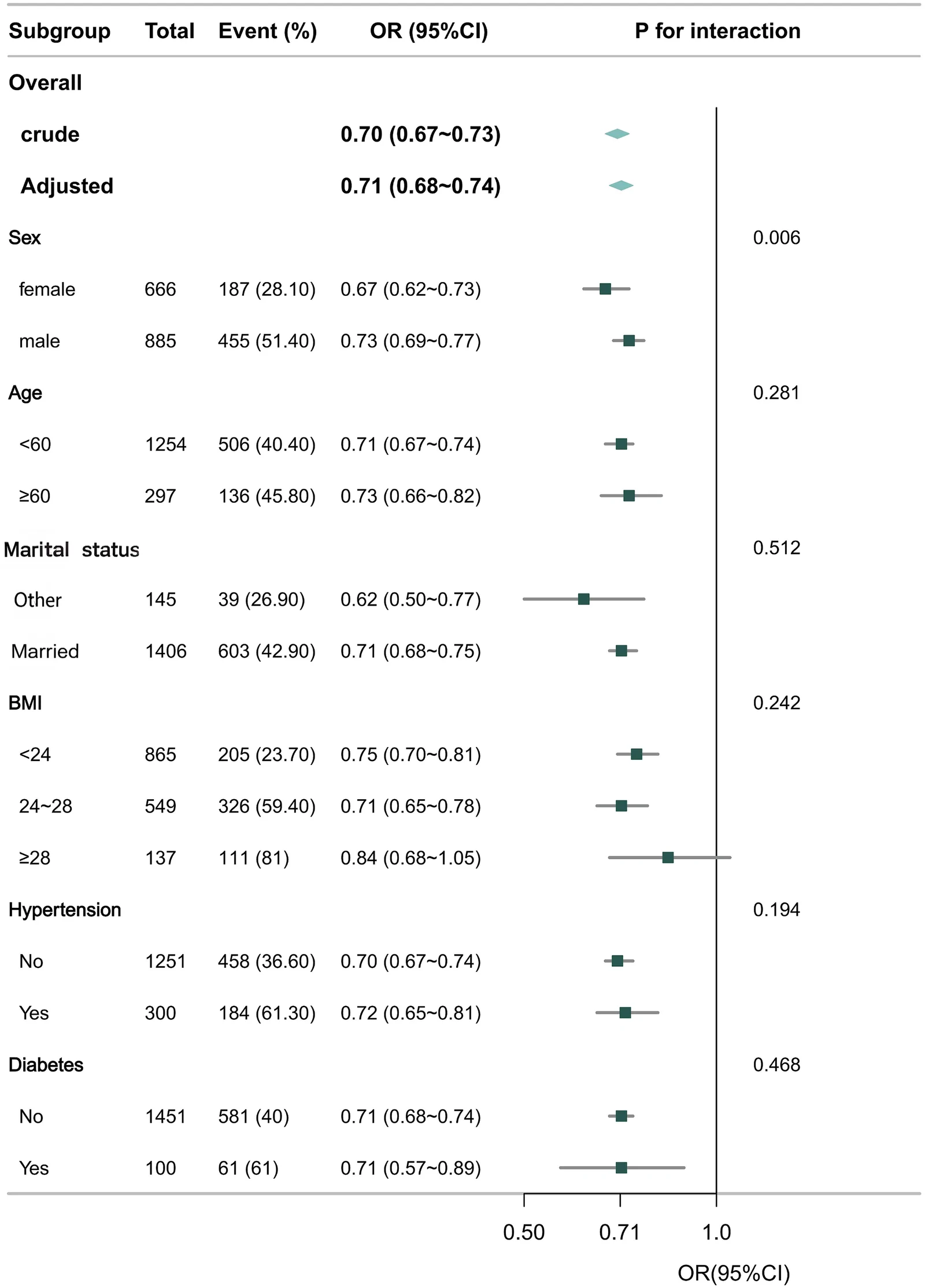
Forest plot graphic summarizing odds ratios with 95 percent confidence intervals for event occurrence across subgroups including sex, age, marital status. BMI, hypertension, and diabetes with adjusted and crude overall values and P values for interaction displayed in columns.

### Sensitivity analyses

Sensitivity analyses yielded findings generally consistent with the primary analysis. The inverse association between NMRI and prevalent MASLD remained evident in the complete-case analysis (**Supplementary Table S6**), after recalculation using hemoglobin-corrected albumin (**Supplementary Table S7**), after excluding participants with extreme BMI (**Supplementary Table S8**) or with hypertension or diabetes (**Supplementary Table S9**), and after removing the upper limit for the actual weight/ideal weight ratio in the GNRI formula (**Supplementary Table S10**).

In the additional analysis using the alternative reduced-overlap outcome, the inverse association with NMRI persisted but was attenuated. In the fully adjusted model, the OR per one-unit increase in NMRI was 0.79 (95% CI, 0.76–0.82; P< 0.001), while the ORs for the second and third tertiles versus the first tertile were 0.33 (95% CI, 0.25–0.43) and 0.12 (95% CI, 0.09–0.17), respectively (P for trend< 0.001; **Supplementary Table S11**). Adding NMRI to the basic model increased the AUC from 0.686 (95% CI, 0.660–0.712) to 0.766 (95% CI, 0.742–0.789; P< 0.001), with an NRI of 0.601 (95% CI, 0.506–0.697) and an IDI of 0.105 (95% CI, 0.090–0.120) (both P< 0.001; **Supplementary Table S12**). These estimates were attenuated relative to those obtained using the primary MASLD definition. Finally, hierarchical clustering showed substantial concordance with the K-means solution (overall agreement, 84.27%; Cohen’s κ=0.7624), supporting the consistency of the statistically derived profiles across clustering methods (**Supplementary Table S13**; **Supplementary Figures S1**, **S2**).

## Discussion

In this retrospective cross-sectional study, higher NMRI was consistently associated with lower odds of prevalent MASLD and showed favorable within-sample discrimination in a sub-plateau health-examination population. The association was strong and approximately linear, suggesting that NMRI captures interrelated nutritional, metabolic, and anthropometric characteristics associated with MASLD status. Three statistically derived metabolic–nutritional profiles were also identified, with marked differences in the odds of prevalent MASLD, further indicating heterogeneity in these characteristics across the study population. NMRI showed higher discrimination than its individual components and improved discrimination, reclassification, and net benefit when added to the basic model. Bootstrap internal validation indicated limited optimism, with the AUC decreasing from 0.822 to 0.815 after optimism correction. Collectively, these findings support further evaluation of NMRI as a parsimonious composite indicator associated with prevalent MASLD.

Our findings build on established evidence that individual metabolic, nutritional, and anthropometric markers, including TyG, GNRI, and WHtR, are associated with MASLD (28–30). Rather than considering these measures solely as isolated correlates, we explored their combined characteristics within the study population. K-means clustering identified three statistically derived profiles: an adiposity predominant profile, an unfavorable metabolic nutritional profile, and a favorable metabolic profile, with substantially different odds of prevalent MASLD.These findings suggest heterogeneity in the clustering of metabolic, anthropometric, and nutrition-related characteristics among individuals with different MASLD status. In this context, the previously proposed NMRI provides a parsimonious means of integrating GNRI, TyG, and WHtR into a single composite measure representing these interrelated dimensions.

Compared with conventional single-domain indices such as the Lipid Accumulation Product (LAP) and other individual metabolic proxies, NMRI incorporates metabolic, anthropometric, and nutritional-related information within a single composite measure, thereby providing a broader representation of these characteristics (31). This multidimensional structure may be particularly relevant in metabolically heterogeneous populations. Nevertheless, simultaneous inclusion of GNRI, TyG, and WHtR as separate predictors yielded numerically higher discriminative performance than NMRI, indicating that the potential value of NMRI lies primarily in its parsimonious integration of these dimensions rather than superiority over separate-component modeling. Given that the present study was conducted in a sub-plateau setting at approximately 1,900 m, we additionally recalculated GNRI and NMRI using hemoglobin-corrected albumin to assess the potential influence of altitude-related hemoconcentration. The association remained similar after this correction, indicating that the findings were not materially altered by this alternative albumin specification.

The observed association between NMRI and MASLD may also be considered within a broader biological framework involving hypoxia and metabolic dysfunction, although these mechanisms were not directly assessed in the present study. Experimental studies suggest that hypoxic conditions can influence hepatic metabolism through hypoxia-inducible factor (HIF) signaling. HIF-2α activation has been associated with hepatic lipid accumulation and impaired fatty-acid oxidation, whereas the contribution of HIF-1α to steatosis appears to vary according to experimental context (32–36). Studies of fatty liver disease have further suggested that lipid-laden hepatocytes may enlarge and distort hepatic sinusoids, potentially impairing microvascular perfusion and contributing to local hypoxia, thereby supporting a possible bidirectional interaction between hepatic lipid accumulation and hypoxia (37). Hypoxic stress may also alter adipose-tissue lipid handling and other metabolic pathways relevant to insulin resistance and hepatic lipid delivery (38, 39).

These mechanisms provide biological context for the observed associations. NMRI is a composite measure of TyG, WHtR, and GNRI and does not directly measure hepatic lipid accumulation, hepatocyte morphology, hepatic microcirculation, tissue oxygenation, or HIF activity. Moreover, the study lacked a lower-altitude comparator and individual-level measures of hypoxic exposure. Accordingly, the sub-plateau altitude should be regarded as a contextual characteristic of the study setting rather than a causal exposure. The larger WQS weights assigned to TyG and WHtR further indicate that metabolic and anthropometric components contributed more strongly to the observed statistical profile than GNRI, but these weights should not be interpreted as evidence for specific hypoxia-related pathways or biological importance. Direct evaluation of hepatic lipid content, tissue oxygenation, microcirculatory function, and molecular hypoxia signaling will be required to investigate these potential mechanisms.

GNRI incorporates serum albumin and body weight and therefore provides nutritional and anthropometric information complementary to the metabolic and adiposity-related components of NMRI; albumin additionally has antioxidant and fatty-acid transport functions (40, 41). However, GNRI alone showed relatively modest discrimination for prevalent MASLD (AUC, 0.618) and was positively associated with MASLD (OR, 1.09; 95% CI, 1.06–1.13). Consistently, its WQS weight (0.19) was smaller than those of TyG (0.41) and WHtR (0.40), suggesting a measurable but comparatively smaller statistical contribution. Importantly, the inverse association of NMRI should also be interpreted in light of its mathematical structure, with GNRI in the numerator and TyG and WHtR in the denominator. Thus, GNRI may provide complementary nutritional and anthropometric information within NMRI.

### Strengths and limitations

This study has several strengths. First, NMRI was evaluated from complementary perspectives, including its association with prevalent MASLD, dose–response pattern, and discriminative and incremental performance. The consistency of the association across multivariable, subgroup, and sensitivity analyses supports the internal consistency of the findings. Second, complementary analytical approaches were applied to characterize different aspects of the study question. Restricted cubic splines assessed the shape of the association, clustering explored metabolic–nutritional heterogeneity, and WQS regression characterized the relative statistical contributions of NMRI components. Third, discriminative performance was assessed using ROC analysis, NRI, IDI, and DCA, while bootstrap resampling quantified optimism in the multivariable model incorporating NMRI. Finally, multiple sensitivity analyses addressed missing data, alternative GNRI calculation, potential altitude-related hemoconcentration, extreme BMI, cardiometabolic conditions, and structural overlap between NMRI components and the primary MASLD definition. Together, these analyses provide a comprehensive assessment of NMRI in this sub-plateau health-examination population.

Several limitations should be acknowledged. First, the retrospective cross-sectional design precludes assessment of temporality, causality, and prognosis; therefore, the findings concern prevalent rather than incident MASLD. Second, partial overlap of TyG and WHtR with the cardiometabolic criteria used to define MASLD may have inflated the observed associations and discrimination, although these findings persisted with attenuation in the reduced-overlap sensitivity analysis. Third, hepatic steatosis was assessed by ultrasonography rather than quantitative imaging or histology, which may have resulted in outcome misclassification, particularly for mild steatosis. Fourth, the single-center health-examination population and absence of independent external validation limit generalizability. In addition, the alcohol exclusion thresholds used in this study (>140 g/week for men and >70 g/week for women) were more restrictive than those in the current MASLD/MetALD nomenclature and may further limit generalizability to individuals with higher alcohol consumption, particularly those within the MetALD spectrum. Moreover, the lack of a lower-altitude comparator and individual-level measures of hypoxic exposure precludes attribution of the findings to altitude or hypoxia. Residual confounding from unmeasured lifestyle, socioeconomic, genetic, and environmental factors also remains possible. Accordingly, prospective multicenter studies with independent external validation and quantitative assessment of hepatic steatosis are needed to establish the temporal, discriminative, and clinical utility of NMRI.

## Conclusion

In this cross-sectional sub-plateau population, higher NMRI was consistently associated with lower odds of prevalent MASLD and showed favorable within-sample discrimination of ultrasound-defined MASLD. As a single composite measure integrating nutritional, metabolic, and anthropometric information, NMRI may provide a parsimonious representation of these interrelated characteristics. However, the present findings do not establish prospective risk prediction or diagnostic accuracy against a definitive reference standard. Prospective, multicenter studies incorporating quantitative assessment of hepatic steatosis are needed to evaluate its temporal, discriminative, and clinical utility.

## Data Availability

The datasets presented in this article are not readily available because Some or all datasets generated during and/or analyzed during the current study are not publicly available but are available from the corresponding author on reasonable request. Requests to access the datasets should be directed to zhoutingyuan_wy@126.com.
