# Supplementary material for: Association of the nutritional metabolic risk index with metabolic dysfunction-associated steatotic liver disease in a sub-plateau population
Source: Front Endocrinol (Lausanne). 2026 Sep 30;17:1952423. doi: 10.3389/fendo.2026.1952423 (PMC13661332; doi:10.3389/fendo.2026.1952423)

Association of the Nutritional Metabolic Risk Index With Metabolic Dysfunction-Associated Steatotic Liver Disease in a Sub-Plateau Population

Exposures

Nutritional-Metabolic Risk Index (NMRI)

TyG WHtR GNRI

Integrates metabolic, anthropometric, and nutritional-related information.

Metabolic– nutritional profiles associated with prevalent MASLD

Cluster 1 Adiposity-predominant profile

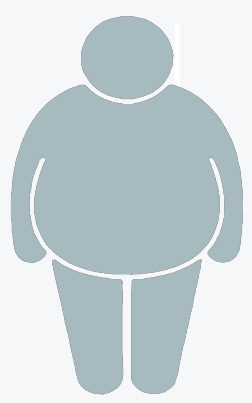

Higher waist-related burden.

Cluster 2 Unfavorable metabolic-nutritional profile

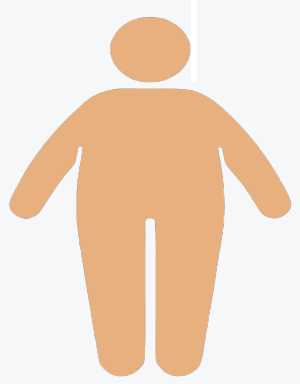

Higher TyG, WHtR, and GNRI

Cluster 3 Favorable metabolic-nutritional profile

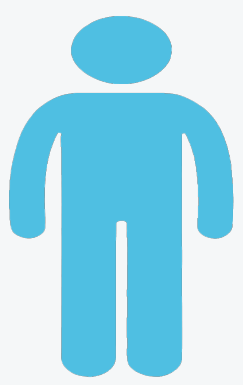

Lower TyG and WHtR.

Methods

Phenotype discovery

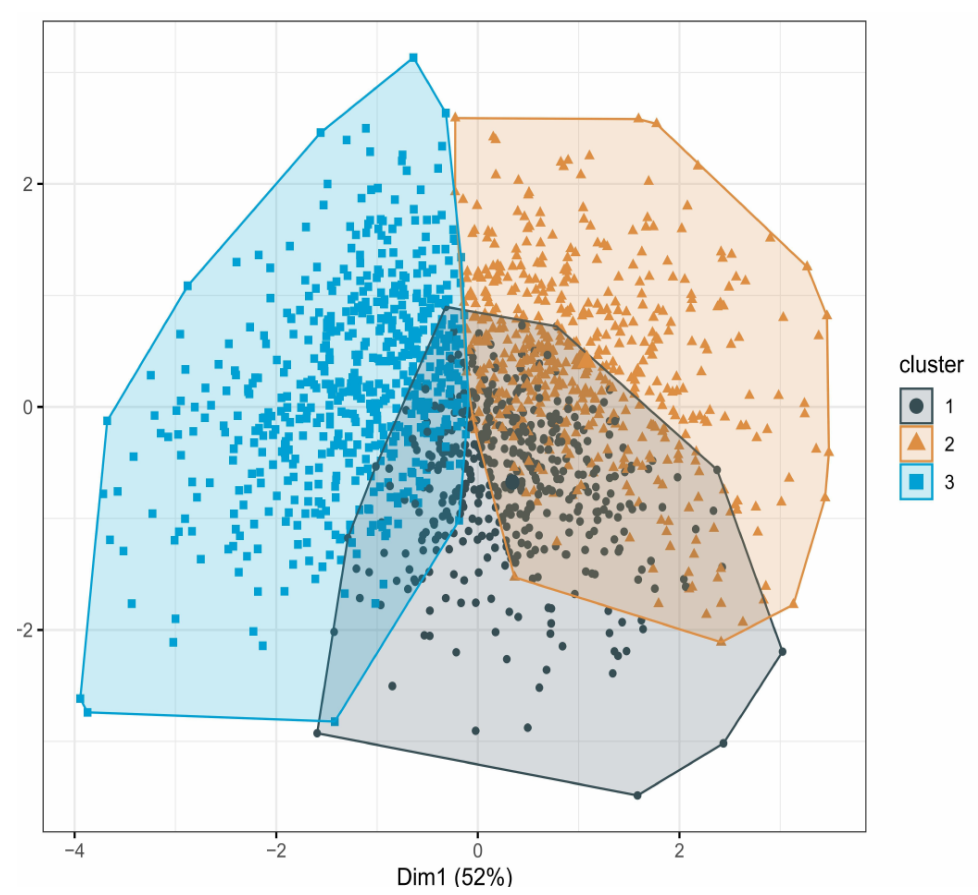

K-means clustering

Cluster distribution

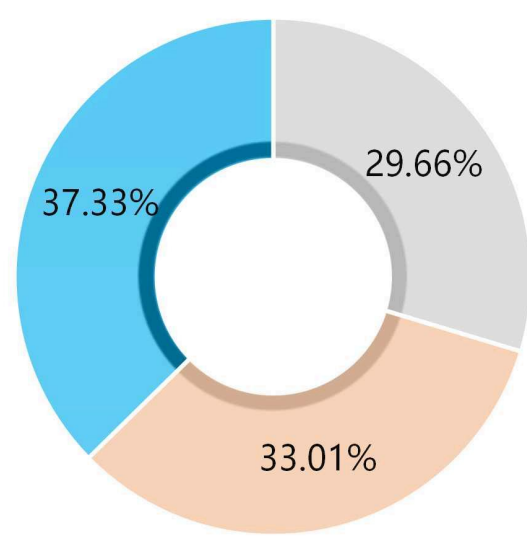

Modeling and validation
Logistic regression | Restricted cubic splines ROC, NRI, IDI and DCA | Subgroup analysis
Weighted quantile sum regression

Mixture contribution analysis

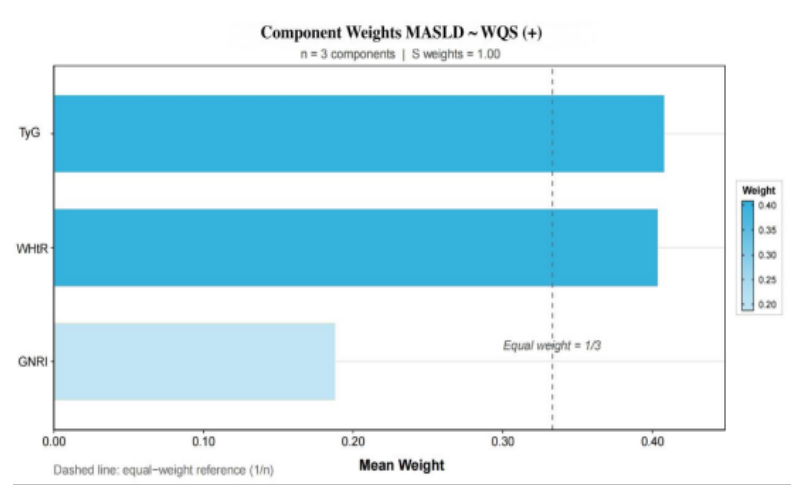

Results

Higher NMRI was linearly associated with lower MASLD risk.

OR = 0.71 AUC = 79.96%

Dose-response relationship

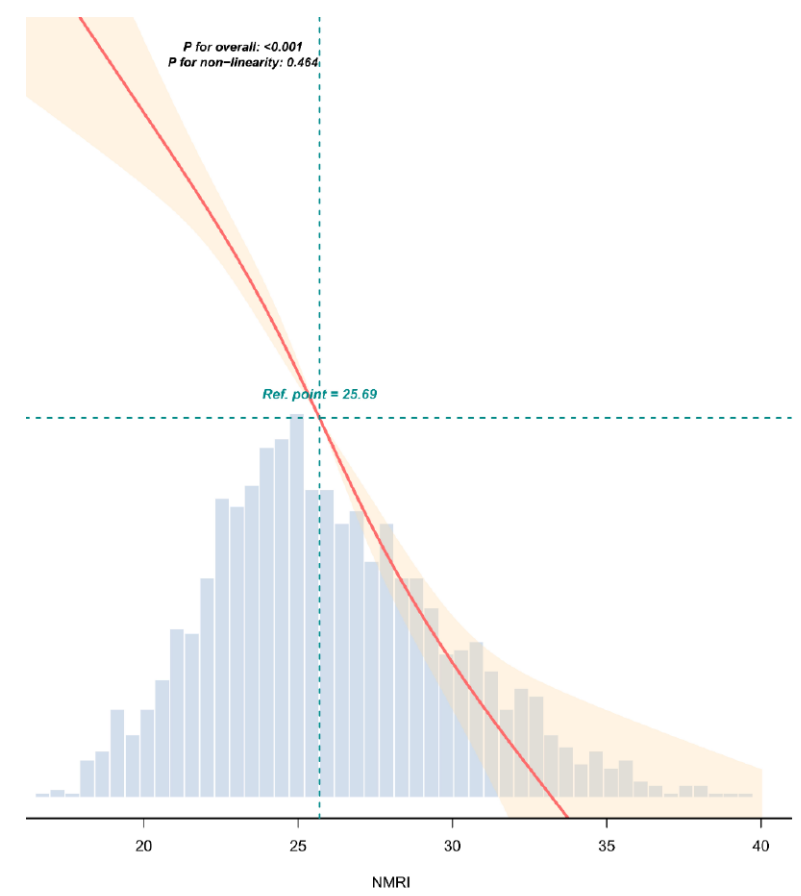

Discrimination performance

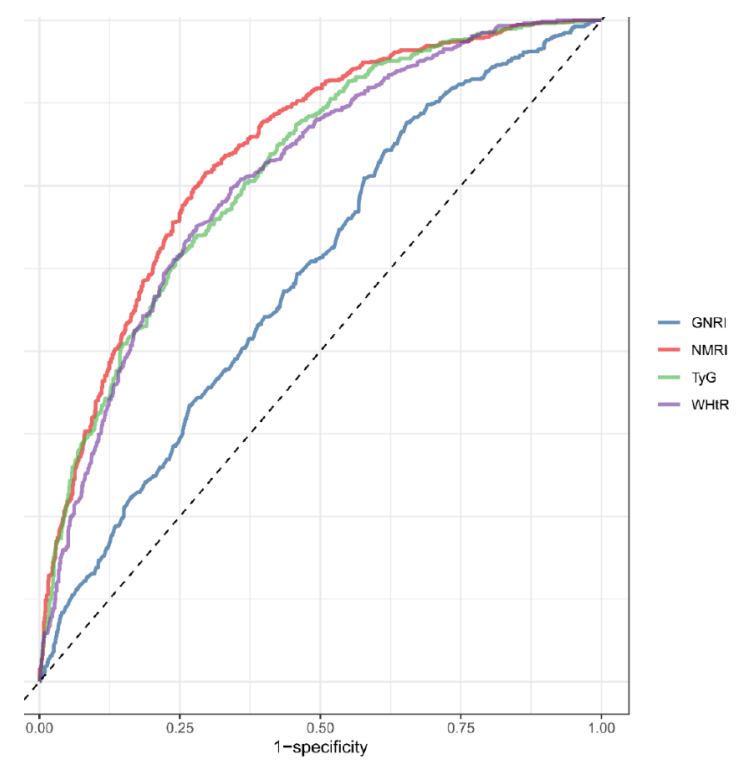

Subgroup consistency

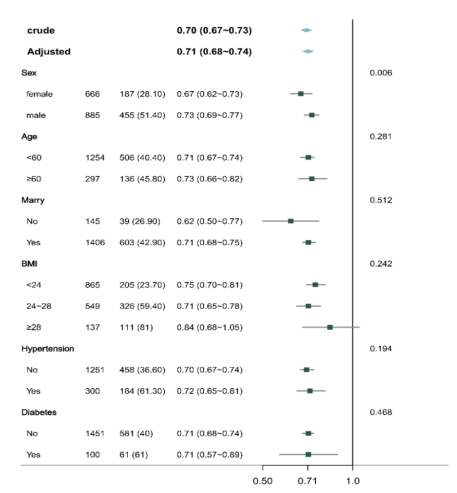

Supplement: Supplementary file 1 [file DataSheet1.pdf]
