# Supplementary material for: Association of the nutritional metabolic risk index with metabolic dysfunction-associated steatotic liver disease in a sub-plateau population
Source: Front Endocrinol (Lausanne). 2026 Sep 30;17:1952423. doi: 10.3389/fendo.2026.1952423 (PMC13661332; doi:10.3389/fendo.2026.1952423)
Supplement: Supplementary file 2 [file DataSheet2.docx]

**Supplementary Materials**

**contents**

[Table S1. Details of missing values.	1](#_Toc12506)

[Table S2. Collinearity Diagnostics for Covariates Based on Tolerance and Variance Inflation Factor (VIF)	2](#_Toc22703)

[Table S3. Baseline characteristics across metabolic–nutritional profiles derived from k-means clustering	3](#_Toc23901)

[Table S4. Receiver operating characteristic (ROC) curve analysis of GNRI, TyG, WHtR, and NMRI for identifying MASLD	4](#_Toc32388)

[Table S5. Bootstrap internal validation of the multivariable model incorporating NMRI for prevalent MASLD	5](#_Toc5578)

[Table S6. Sensitivity analysis excluding participants with missing covariate data in the overall sample	5](#_Toc29053)

[Table S7. Sensitivity analysis using Altitude-Adjusted Hemoglobin-Corrected Albumin	6](#_Toc1218)

[Table S8. Sensitivity analysis after excluding BMI <18.5 or ≥ 35 kg/m²	7](#_Toc30473)

[Table S9. Sensitivity analysis after excluding participants with hypertension, diabetes	8](#_Toc32354)

[Table S10. Sensitivity analysis using the original raw weight ratio in GNRI	9](#_Toc26235)

[Table S11. Sensitivity analysis of the association between NMRI and an alternative reduced-overlap outcome	10](#_Toc10675)

[Table S12. Incremental discriminative performance of NMRI for the alternative reduced-overlap outcome	11](#_Toc19381)

[Table S13. Internal diagnostics and cross-method assessment of the three-cluster solution	12](#_Toc18464)

[Figure S1. The elbow plots for both K-means and hierarchical clustering.	14](#_Toc31550)

[Figure S2. Visualization and clinical characteristics of the three clusters derived by hierarchical clustering](#_Toc14774)**[.](#_Toc14774)** [.	1](#_Toc14774)5

Table S1. Details of missing values.

| **Variable** | **Total sample, n** | **Missing, n** | **Missing, %** |
| --- | --- | --- | --- |
| Age | 1551 | 0 | 0% |
| Sex | 1551 | 0 | 0% |
| Marital status | 1551 | 215 | 13.86% |
| Smoking | 1551 | 185 | 11.93% |
| Drinking | 1551 | 185 | 11.93% |
| Hypertension | 1551 | 1 | 0.06% |
| Diabetes | 1551 | 1 | 0.06% |
| ALT | 1551 | 79 | 5.09% |
| AST | 1551 | 79 | 5.09% |
| LDL-C | 1551 | 0 | 0% |
| hs-CRP | 1551 | 84 | 5.42% |
| BUN | 1551 | 80 | 5.16% |

Abbreviations: ALT: alanine aminotransferase; AST: aspartate aminotransferase; LDL-C: low-density lipoprotein cholesterol; hs-CRP: high-sensitivity C-reactive protein; BUN: blood urea nitrogen.

Table S2. Collinearity Diagnostics for Covariates Based on Tolerance and Variance Inflation Factor (VIF)

| **Variables** | **Df** | **VIF** |
| --- | --- | --- |
| Age | 1 | 1.879 |
| Sex | 1 | 2.644 |
| Marital status | 1 | 1.142 |
| Smoking status | 1 | 1.399 |
| Drinking status | 1 | 1.638 |
| Hypertension | 1 | 1.258 |
| Diabetes | 1 | 1.22 |
| LDL-C | 1 | 1.056 |
| ALT | 1 | 2.699 |
| AST | 1 | 2.586 |
| BUN | 1 | 1.192 |
| Hs-CRP | 1 | 1.044 |

Abbreviations: ALT: alanine aminotransferase; AST: aspartate aminotransferase; LDL-C: low-density lipoprotein cholesterol; hs-CRP: high-sensitivity C-reactive protein; BUN: blood urea nitrogen. All adjusted VIFs were well below commonly used thresholds for concern (e.g., VIF ≥ 5), indicating no meaningful multicollinearity among covariates. Accordingly, all listed variables were considered suitable for inclusion in subsequent multivariable analyses without further collinearity-driven adjustment.

Table S3. Baseline characteristics across metabolic–nutritional profiles derived from k-means clustering

| **Variables** | **Total**  **(n = 1551)** | **Cluster 1**  **(n = 460)** | **Cluster 2**  **(n = 512)** | **Cluster 3**  **(n = 579)** | **p** |
| --- | --- | --- | --- | --- | --- |
| Age (years) | 48.77 ± 12.19 | 53.52 ± 11.43 | 48.42 ± 11.48 | 45.30 ± 12.18 | < 0.001 |
| Sex, n (%) |  |  |  |  | < 0.001 |
| Female | 666 (42.94) | 194 (42.17) | 137 (26.76) | 335 (57.86) |  |
| Male | 885 (57.06) | 266 (57.83) | 375 (73.24) | 244 (42.14) |  |
| Marital status, n (%) |  |  |  |  | < 0.001 |
| Other | 145 ( 9.35) | 13 (2.83) | 51 (9.96) | 81 (13.99) |  |
| Married | 1406 (90.65) | 447 (97.17) | 461 (90.04) | 498 (86.01) |  |
| Smoking status, n (%) |  |  |  |  | < 0.001 |
| NO | 1221 (78.72) | 355 (77.17) | 372 (72.66) | 494 (85.32) |  |
| Yes | 330 (21.28) | 105 (22.83) | 140 (27.34) | 85 (14.68) |  |
| Drinking status, n (%) |  |  |  |  | < 0.001 |
| NO | 1056 (68.09) | 311 (67.61) | 292 (57.03) | 453 (78.24) |  |
| Yes | 495 (31.91) | 149 (32.39) | 220 (42.97) | 126 (21.76) |  |
| Hypertension, n (%) |  |  |  |  | < 0.001 |
| NO | 1251 (80.66) | 334 (72.61) | 379 (74.02) | 538 (92.92) |  |
| Yes | 300 (19.34) | 126 (27.39) | 133 (25.98) | 41 (7.08) |  |
| Diabetes, n (%) |  |  |  |  | < 0.001 |
| NO | 1451 (93.55) | 432 (93.91) | 450 (87.89) | 569 (98.27) |  |
| Yes | 100 ( 6.45) | 28 (6.09) | 62 (12.11) | 10 (1.73) |  |
| BMI, (kg/m²) | 23.77 ± 3.04 | 25.40 ± 2.77 | 24.88 ± 2.64 | 21.50 ± 2.04 | < 0.001 |
| FBG (mmol/L) | 5.04 ± 1.28 | 5.06 ± 1.12 | 5.52 ± 1.72 | 4.61 ± 0.65 | < 0.001 |
| TC (mmol/L) | 4.86 ± 0.95 | 4.85 ± 0.95 | 5.11 ± 0.99 | 4.65 ± 0.86 | < 0.001 |
| TG (mmol/L) | 1.51 (1.07, 2.20) | 1.59 (1.24, 2.12) | 2.20 (1.64, 3.18) | 1.05 (0.82, 1.39) | < 0.001 |
| HDL-C (mmol/L) | 1.29 ± 0.33 | 1.23 ± 0.29 | 1.16 ± 0.29 | 1.45 ± 0.34 | < 0.001 |
| LDL-C (mmol/L) | 2.92 ± 0.81 | 2.97 ± 0.83 | 3.00 ± 0.85 | 2.80 ± 0.74 | < 0.001 |
| ALT (U/L) | 20.10 (13.60, 30.67) | 19.80 (13.57, 28.50) | 26.00 (18.28, 38.62) | 16.50 (11.50, 24.45) | < 0.001 |
| AST (U/L) | 27.65 ± 9.78 | 27.08 ± 9.03 | 30.00 ± 11.84 | 26.02 ± 7.74 | < 0.001 |
| Creatinine (μmol/L) | 80.17 ± 16.64 | 80.46 ± 18.25 | 83.06 ± 14.88 | 77.38 ± 16.34 | < 0.001 |
| BUN (mmol/L) | 5.17 ± 1.27 | 5.20 ± 1.29 | 5.29 ± 1.27 | 5.04 ± 1.23 | 0.004 |
| hs-CRP (mg/L) | 3.44 (0.92, 5.03) | 2.38 (0.91, 4.83) | 3.10 (0.85, 5.01) | 3.63 (1.02, 5.12) | 0.18 |
| GNRI, Mean ± SD | 110.74 ± 4.03 | 108.95 ± 2.59 | 114.28 ± 2.70 | 109.03 ± 3.91 | < 0.001 |
| TyG, Mean ± SD | 8.75 ± 0.66 | 8.80 ± 0.47 | 9.23 ± 0.65 | 8.27 ± 0.43 | < 0.001 |
| WHtR, Mean ± SD | 0.49 ± 0.05 | 0.54 ± 0.04 | 0.51 ± 0.04 | 0.44 ± 0.03 | < 0.001 |

Abbreviations: BMI, body mass index; FBG: fasting blood glucose; TC: total cholesterol; TG: triglycerides; HDL-C: high-density lipoprotein cholesterol; LDL-C: low-density lipoprotein cholesterol; ALT: alanine aminotransferase; AST: aspartate aminotransferase; BUN: blood urea nitrogen; hs-CRP: high-sensitivity C-reactive protein; GNRI: geriatric nutritional risk index; TyG: triglyceride-glucose index; WHtR: waist-height ratio; NMRI: Nutritional Metabolic Risk Index.

Table S3 details the baseline characteristics of the three clusters identified by k-means analysis. Cluster 1 (n = 460) was characterized by an adiposity-predominant profile, with the highest mean waist-to-height ratio (WHtR: 0.54 ± 0.04) and a relatively lower mean triglyceride-glucose index (TyG: 8.80 ± 0.47). Cluster 2 (n = 512) showed an unfavorable metabolic–nutritional profile, characterized by higher GNRI, WHtR, and TyG values, together with the highest BMI, fasting blood glucose, triglyceride levels, and prevalences of diabetes and hypertension. In contrast, Cluster 3 (n = 579) showed a comparatively favorable metabolic profile, characterized by lower WHtR, TyG, BMI, fasting blood glucose, and triglyceride levels, lower prevalences of metabolic conditions, and higher HDL-C. High-sensitivity C-reactive protein levels did not differ significantly across clusters (P = 0.18).

Table S4. Receiver operating characteristic (ROC) curve analysis of GNRI, TyG, WHtR, and NMRI for identifying MASLD

| **Variables** | **Cut-off value** | **Sensitivity**  **(%)** | **Specificity**  **(%)** | **Youden index (%)** | **AUC(95%CI)(%)** | **Z** | **P** |
| --- | --- | --- | --- | --- | --- | --- | --- |
| GNRI | 107.51 | 84.58 | 34.65 | 19.23 | 61.82(59.03 ~ 64.60) | 8.32 | <0.001 |
| TyG | 8.21 | 67.13 | 72.50 | 39.63 | 77.37 (75.08 ~ 79.67) | 23.37 | <0.001 |
| WHtR | 0.45 | 68.85 | 72.06 | 40.91 | 76.43 (74.08 ~ 78.78) | 22.04 | <0.001 |
| NMRI | 22.78 | 76.95 | 70.41 | 47.36 | 79.96 (77.76 ~ 82.14) | 26.81 | <0.001 |

Abbreviations: GNRI, Geriatric Nutritional Risk Index; TyG, triglyceride–glucose index; WHtR, waist-to-height ratio; NMRI, Nutritional Metabolic Risk Index; MASLD, metabolic dysfunction-associated steatotic liver disease; AUC, area under the receiver operating characteristic curve; CI, confidence interval.

Optimal cut-off values were determined using the Youden index. Sensitivity and specificity are expressed as percentages. Z denotes the test statistic for testing the null hypothesis that AUC = 0.5. All tests were two-sided, and P < 0.05 was considered statistically significant.

Table S5. Bootstrap internal validation of the multivariable model incorporating NMRI for prevalent MASLD

| **Performance measure** | **Estimate** |
| --- | --- |
| Apparent AUC | 0.822 |
| Mean bootstrap optimism | 0.007 |
| SD of bootstrap optimism | 0.011 |
| 2.5th–97.5th percentile of bootstrap optimism | −0.013 to 0.028 |
| Optimism-corrected AUC | 0.815 |

Internal validation was performed using 1,000 bootstrap resamples. The logistic regression model included NMRI, age, sex, marital status, smoking status, drinking status, hypertension, diabetes, LDL-C, ALT, AST, BUN, and hs-CRP. In each bootstrap resample, the model was refitted and its AUC was evaluated in both the bootstrap sample and the original dataset. Optimism was calculated as the mean difference between these AUCs across the 1,000 resamples, and the optimism-corrected AUC was obtained by subtracting the mean optimism from the apparent AUC.

Table S6 Sensitivity analysis excluding participants with missing covariate data in the overall sample

| **Exposure** | **Crude model** | | **Model 1** | | **Model 2** | |
| --- | --- | --- | --- | --- | --- | --- |
|  | **OR (95% CI)** | ***P* value** | **OR (95% CI)** | ***P* value** | **OR (95% CI)** | ***P* value** |
| NMRI | 0.69 (0.65~0.72) | <0.001 | 0.68 (0.65~0.72) | <0.001 | 0.7 (0.66~0.74) | <0.001 |
| NMRI tertile |  |  |  |  |  |  |
| T1 | 1(Ref) |  | 1(Ref) |  | 1(Ref) |  |
| T2 | 0.28 (0.2~0.39) | <0.001 | 0.28 (0.2~0.39) | <0.001 | 0.3 (0.21~0.42) | <0.001 |
| T3 | 0.05 (0.03~0.07) | <0.001 | 0.05 (0.03~0.08) | <0.001 | 0.06 (0.04~0.1) | <0.001 |
| P for trend |  | <0.001 |  | <0.001 |  | <0.001 |

Data presented are ORs and 95% CIs. Model 1: adjusted for age, sex, marital status, smoking status, and drinking status. Model 2: adjusted for variables in Model 1 plus hypertension, diabetes, LDL-C, ALT, AST, BUN, and hs-CRP. Abbreviations: NMRI: Nutritional Metabolic Risk Index; LDL-C: low-density lipoprotein cholesterol; ALT: alanine aminotransferase; AST: aspartate aminotransferase; BUN: blood urea nitrogen; hs-CRP: high-sensitivity C-reactive protein.

Table S7 Sensitivity analysis using Altitude-Adjusted Hemoglobin-Corrected Albumin

| **Exposure** | **Crude model** | | **Model 1** | | **Model 2** | |
| --- | --- | --- | --- | --- | --- | --- |
|  | **OR (95% CI)** | ***P* value** | **OR (95% CI)** | ***P* value** | **OR (95% CI)** | ***P* value** |
| NMRI | 0.7 (0.67~0.73) | <0.001 | 0.69 (0.66~0.72) | <0.001 | 0.71 (0.68~0.74) | <0.001 |
| NMRI tertile |  |  |  |  |  |  |
| T1 | 1(Ref) |  | 1(Ref) |  | 1(Ref) |  |
| T2 | 0.3 (0.24~0.39) | <0.001 | 0.29 (0.22~0.38) | <0.001 | 0.31 (0.23~0.41) | <0.001 |
| T3 | 0.06 (0.04~0.08) | <0.001 | 0.06 (0.04~0.08) | <0.001 | 0.07 (0.05~0.11) | <0.001 |
| P for trend |  | <0.001 |  | <0.001 |  | <0.001 |

Data presented are ORs and 95% CIs. Model 1: adjusted for age, sex, marital status, smoking status, and drinking status. Model 2: adjusted for variables in Model 1 plus hypertension, diabetes, LDL-C, ALT, AST, BUN, and hs-CRP. Abbreviations: NMRI: Nutritional Metabolic Risk Index; LDL-C: low-density lipoprotein cholesterol; ALT: alanine aminotransferase; AST: aspartate aminotransferase; BUN: blood urea nitrogen; hs-CRP: high-sensitivity C-reactive protein.

Corrected albumin = measured albumin × (target hemoglobin / measured hemoglobin). Target hemoglobin: 151 g/L for males, 131 g/L for females, based on WHO 2024 sea-level references (140 g/L for males, 120 g/L for females) plus 11 g/L altitude adjustment for 1500–1999 m. GNRI and NMRI were recalculated accordingly.

Table S8 Sensitivity analysis after excluding BMI <18.5 or ≥ 35 kg/m²

| **Exposure** | **Crude model** | | **Model 1** | | **Model 2** | |
| --- | --- | --- | --- | --- | --- | --- |
|  | **OR (95% CI)** | ***P* value** | **OR (95% CI)** | ***P* value** | **OR (95% CI)** | ***P* value** |
| NMRI | 0.7 (0.67~0.73) | <0.001 | 0.69 (0.66~0.73) | <0.001 | 0.71 (0.68~0.75) | <0.001 |
| NMRI tertile |  |  |  |  |  |  |
| T1 | 1(Ref) |  | 1(Ref) |  | 1(Ref) |  |
| T2 | 0.32 (0.24~0.41) | <0.001 | 0.3 (0.23~0.4) | <0.001 | 0.33 (0.25~0.43) | <0.001 |
| T3 | 0.07 (0.05~0.09) | <0.001 | 0.06 (0.05~0.09) | <0.001 | 0.08 (0.06~0.12) | <0.001 |
| P for trend |  | <0.001 |  | <0.001 |  | <0.001 |

Data presented are ORs and 95% CIs. Model 1: adjusted for age, sex, marital status, smoking status, and drinking status. Model 2: adjusted for variables in Model 1 plus hypertension, diabetes, LDL-C, ALT, AST, BUN, and hs-CRP. Abbreviations: NMRI: Nutritional Metabolic Risk Index; LDL-C: low-density lipoprotein cholesterol; ALT: alanine aminotransferase; AST: aspartate aminotransferase; BUN: blood urea nitrogen; hs-CRP: high-sensitivity C-reactive protein.

Table S9 Sensitivity analysis after excluding participants with hypertension and diabetes

| **Exposure** | **Crude model** | | **Model 1** | | **Model 2** | |
| --- | --- | --- | --- | --- | --- | --- |
|  | **OR (95% CI)** | ***P* value** | **OR (95% CI)** | ***P* value** | **OR (95% CI)** | ***P* value** |
| NMRI | 0.7 (0.67~0.73) | <0.001 | 0.7 (0.66~0.73) | <0.001 | 0.71 (0.67~0.75) | <0.001 |
| NMRI tertile |  |  |  |  |  |  |
| T1 | 1(Ref) |  | 1(Ref) |  | 1(Ref) |  |
| T2 | 0.26 (0.19~0.34) | <0.001 | 0.24 (0.18~0.33) | <0.001 | 0.26 (0.19~0.36) | <0.001 |
| T3 | 0.05 (0.03~0.08) | <0.001 | 0.05 (0.04~0.08) | <0.001 | 0.06 (0.04~0.1) | <0.001 |
| P for trend |  | <0.001 |  | <0.001 |  | <0.001 |

Data presented are ORs and 95% CIs. Model 1: adjusted for age, sex, marital status, smoking status, and drinking status. Model 2: adjusted for variables in Model 1 plus LDL-C, ALT, AST, BUN, and hs-CRP. Abbreviations: NMRI: Nutritional Metabolic Risk Index; LDL-C: low-density lipoprotein cholesterol; ALT: alanine aminotransferase; AST: aspartate aminotransferase; BUN: blood urea nitrogen; hs-CRP: high-sensitivity C-reactive protein.

Table S10 Sensitivity analysis using the original raw weight ratio in GNRI

| **Exposure** | **Crude model** | | **Model 1** | | **Model 2** | |
| --- | --- | --- | --- | --- | --- | --- |
|  | **OR (95% CI)** | ***P* value** | **OR (95% CI)** | ***P* value** | **OR (95% CI)** | ***P* value** |
| NMRI | 0.7 (0.67~0.73) | <0.001 | 0.69 (0.66~0.72) | <0.001 | 0.71 (0.68~0.74) | <0.001 |
| NMRI tertile |  |  |  |  |  |  |
| T1 | 1(Ref) |  | 1(Ref) |  | 1(Ref) |  |
| T2 | 0.3 (0.24~0.39) | <0.001 | 0.29 (0.22~0.38) | <0.001 | 0.31 (0.23~0.41) | <0.001 |
| T3 | 0.06 (0.04~0.08) | <0.001 | 0.06 (0.04~0.08) | <0.001 | 0.07 (0.05~0.11) | <0.001 |
| P for trend |  | <0.001 |  | <0.001 |  | <0.001 |

Data presented are ORs and 95% CIs. Model 1: adjusted for age, sex, marital status, smoking status, and drinking status. Model 2: adjusted for variables in Model 1 plus hypertension, diabetes, LDL-C, ALT, AST, BUN, and hs-CRP. Abbreviations: NMRI: Nutritional Metabolic Risk Index; LDL-C: low-density lipoprotein cholesterol; ALT: alanine aminotransferase; AST: aspartate aminotransferase; BUN: blood urea nitrogen; hs-CRP: high-sensitivity C-reactive protein.

The upper limit of 1 for the actual weight/ideal weight ratio in the GNRI formula was removed. GNRI and NMRI were recalculated using the original raw ratio without ceiling restriction.

Table S11 Sensitivity analysis of the association between NMRI and an alternative reduced-overlap outcome

| **Exposure** | **Crude model** | | **Model 1** | | **Model 2** | |
| --- | --- | --- | --- | --- | --- | --- |
|  | **OR (95% CI)** | ***P* value** | **OR (95% CI)** | ***P* value** | **OR (95% CI)** | ***P* value** |
| NMRI | 0.79 (0.76~0.81) | <0.001 | 0.78 (0.75~0.81) | <0.001 | 0.79 (0.76~0.82) | <0.001 |
| NMRI tertile |  |  |  |  |  |  |
| T1 | 1(Ref) |  | 1(Ref) |  | 1(Ref) |  |
| T2 | 0.32 (0.25~0.42) | <0.001 | 0.31 (0.24~0.4) | <0.001 | 0.33 (0.25~0.43) | <0.001 |
| T3 | 0.12 (0.09~0.16) | <0.001 | 0.11 (0.08~0.15) | <0.001 | 0.12 (0.09~0.17) | <0.001 |
| P for trend |  | <0.001 |  | <0.001 |  | <0.001 |

Data presented are ORs and 95% CIs. Model 1: adjusted for age, sex, marital status, smoking status, and drinking status. Model 2: adjusted for variables in Model 1 plus hypertension, diabetes, LDL-C, ALT, AST, BUN, and hs-CRP.

The alternative reduced-overlap outcome was defined as ultrasound-confirmed hepatic steatosis together with elevated blood pressure and/or low HDL-C, with adiposity-, glucose-, and triglyceride-based cardiometabolic criteria excluded to minimize direct overlap with NMRI components.

Abbreviations: NMRI: Nutritional Metabolic Risk Index; LDL-C: low-density lipoprotein cholesterol; ALT: alanine aminotransferase; AST: aspartate aminotransferase; BUN: blood urea nitrogen; hs-CRP: high-sensitivity C-reactive protein.

Table S12 Incremental discriminative performance of NMRI for the alternative reduced-overlap outcome

| **Model** | **AUC (95% CI)** | ***P-*value** | **NRI (95% CI)** | ***P-*value** | **IDI (95% CI)** | ***P-*value** |
| --- | --- | --- | --- | --- | --- | --- |
| Basic model | 0.686 (0.660, 0.712) |  | Ref |  | Ref |  |
| +NMRI | 0.766 (0.742, 0.789) | <0.001 | 0.601(0.506,0.697) | <0.001 | 0.105(0.090,0.120) | <0.001 |

Basic models were adjusted for age, sex, marital status, smoking status, drinking status, hypertension, diabetes, LDL-C, ALT, AST, BUN, and hs-CRP.

The alternative reduced-overlap outcome was defined as ultrasound-confirmed hepatic steatosis together with elevated blood pressure and/or low HDL-C, with adiposity-, glucose-, and triglyceride-based cardiometabolic criteria excluded to minimize direct overlap with NMRI components.

Abbreviations: AUC: Area Under the Curve; NRI: Net reclassification improvement; IDI: Integrated discrimination improvement; NMRI: Nutritional metabolic risk index.

Table S13. Internal diagnostics and cross-method assessment of the three-cluster solution

| **Domain** | **Metric** | **Result** |
| --- | --- | --- |
| Data preprocessing | Variables used for clustering | GNRI, WHtR, and TyG |
|  | Standardization | Z-score standardization |
|  | Distance metric | Euclidean distance |
|  | Total sample size | 1,551 |
| Cluster tendency | Hopkins statistic | 0.822 |
| Cluster-number selection | Candidate solutions evaluated | K = 2–10 |
|  | Primary criterion | Silhouette coefficient, elbow method, BSS/TSS |
|  | Selected number of clusters | K = 3 |
|  | WSS for K = 3 | 2,622.21 |
|  | BSS/TSS for K = 3 | 43.61% |
|  | Average silhouette coefficient for K = 3 | 0.240 |
| Cluster centers and silhouette statistics | Cluster 1, n (%) | 460 (29.7%) |
|  | Standardized center, | GNRI: −0.444, WHtR: 0.809, TyG: 0.077 |
|  | Cluster-specific average silhouette coefficient | 0.282 |
|  | Cluster 2, n (%) | 512 (33.0%) |
|  | Standardized center, | GNRI: 0.878, WHtR: 0.330, TyG: 0.738 |
|  | Cluster-specific average silhouette coefficient | 0.179 |
|  | Cluster 3, n (%) | 579 (37.3%) |
|  | Standardized center, | GNRI: −0.424, WHtR: −0.935, TyG: −0.714 |
|  | Cluster-specific average silhouette coefficient | 0.261 |
| Cross-method concordance | Alternative clustering method | Hierarchical clustering |
|  | Overall observed agreement | 84.27% |
|  | Agreement expected by chance | 33.80% |
|  | Cohen’s κ | 0.7624 |
|  | Cluster 1 concordance | 385/460 (83.7%) |
|  | Cluster 2 concordance | 382/512 (74.6%) |
|  | Cluster 3 concordance | 540/579 (93.3%) |

**Abbreviations:** BSS, between-cluster sum of squares; GNRI, Geriatric Nutritional Risk Index; NMRI, Nutritional Metabolic Risk Index; TSS, total sum of squares; TyG, triglyceride-glucose index; WHtR, waist-to-height ratio; WSS, within-cluster sum of squares.

**Footnote:** The primary clustering analysis was performed using K-means clustering. Hierarchical clustering was used as an alternative clustering approach to assess cross-method concordance. Cluster labels from hierarchical clustering were aligned with the corresponding K-means profiles before agreement was calculated.

Figure S1 The elbow plots for both K-means and hierarchical clustering.

The Elbow plots for both K-means (A) and hierarchical clustering (B) showed a pronounced decrease in WSS with increasing k and an inflection at k = 3 (vertical reference at k = 3), indicating that a three-cluster solution adequately balances model parsimony and within-cluster homogeneity. The concordance between partitioning and hierarchical solutions supports a stable three-group structure in the dataset.These empirically derived clusters were retained for subsequent analyses examining their associations with prevalent MASLD.

**Figure S2. Visualization and clinical characteristics of the three clusters derived by hierarchical clustering.** (A) Cluster distribution of participants. (B) Scaled boxplots. (C) Heatmap. (D) Radar plots. Hierarchical clustering identified three statistically derived groups comprising 42.36%, 31.14%, and 26.50% of participants, respectively. Visual inspection of the boxplots, heatmap, and radar plots showed broadly coherent patterns across the three groups. Cluster 1 was characterized by a favorable metabolic profile, Cluster 2 by an adiposity-predominant profile, and Cluster 3 by an unfavorable metabolic–nutritional profile.
